# The expression of circadian clock genes in *Daphnia magna* diapause

**DOI:** 10.1038/s41598-020-77065-3

**Published:** 2020-11-16

**Authors:** Anke Schwarzenberger, Luxi Chen, Linda C. Weiss

**Affiliations:** 1 Limnological Institute, University of Konstanz, Mainaustraße 252, 78464 Konstanz, Germany; 2 Department of Animal Ecology, Evolution and Biodiversity, Faculty of Biology and Biotechnology, Ruhr University Bochum, Universitaetsstraße 150, 44780 Bochum, Germany

**Keywords:** Developmental biology, Ecology, Limnology

## Abstract

Diapause is a mechanism necessary for survival in arthropods. Often diapause induction and resurrection is light-dependent and therefore dependent on the photoperiod length and on the number of consecutive short-days. In many organisms, including the microcrustacean *Daphnia magna*, one functional entity with the capacity to measure seasonal changes in day-length is the circadian clock. There is a long-standing discussion that the circadian clock also controls photoperiod-induced diapause. We tested this hypothesis in *D. magna*, an organism which goes into a state of suspended animation with the shortening of the photoperiod. We measured gene expression of clock genes in diapause-destined embryos of *D. magna* in the initiation, resting and resurrection phases and checked it against gene expression levels of continuously developing embryos. We demonstrate that some genes of the clock are differentially expressed during diapause induction but not during its maintenance. Furthermore, the photoreceptor gene *cry2* and the clock-associated gene *brp* are highly expressed during induction and early diapause, probably in order to produce excess mRNA to prepare for immediate resurrection. After resurrection, both types of embryos show a similar pattern of gene expression during development. Our study contributes significantly to the understanding of the molecular basis of diapause induction, maintenance and termination.

## Introduction

Diapause is widespread in insects and crustaceans and has undoubtedly contributed to their enormous ecological and evolutionary success by allowing them to exploit resources in favourable seasons and to evade cold winters, desiccation, starvation, predators and parasites (reviewed^1^). The crustacean *Daphnia* is a keystone organism in the carbon transfer from primary producers to secondary consumers, and is a model organism in genetic and (eco)toxicological studies. In *Daphnia*, diapause is a phenotypically plastic trait which is dependent on environmental conditions^2^. Factors inducing diapause in *Daphnia* include food availability, high density of conspecifics, photoperiod, low temperature, predation and desiccation^3–6^. Cyclical parthenogenetic *Daphnia* females can switch from asexual to sexual reproduction in order to produce resting stages (ephippia containing up to two diapausing eggs^5^) that can persist in lake sediments and be resurrected after years or even decades (*cf.*^7–9^).

The regulation of diapause is an intriguing developmental problem, because development is brought to a halt before being resumed a long time later. Diapause in arthropods can be categorized into three different phases: Induction, maintenance and termination^10^; the molecular signals and biochemical mechanisms that drive development through these phases are only partly understood^1^.

Growth, development and metabolism are also arrested in crustaceans during diapause, while tolerance to environmental and physiological stress is increased^11^. In order to maintain this state, a specific pattern of differentially expressed genes is governed (reviewed in^12,13^): The stress-inducible transcription co-factor p8 is up-regulated in the crustacean *Artemia franciscana* both in the induction and in the maintenance of diapause. This is also the case for three small heat shock proteins which might promote diapause maintenance by enhancing stress tolerance. Furthermore, genes that suggest hormonal influence on *Artemia* diapause (i.e. genes that are involved in metabolism or that inhibit cell growth and division) are differentially expressed. Also a low intracellular pH was discussed as being a possible mechanism that inhibits metabolism in dormant cysts of *Artemia*^14^. Specifically for *D. magna*, Pauwels et al*.*^15^ observed higher levels of glycerol and a heat shock protein in dormant than in parthenogenetic eggs.

Photoperiodic induction of winter diapause requires a mechanism for measuring day-length (a clock) and a mechanism for counting the number of short days (a counter). Two rhythms of light exist on earth: The daily rhythm due to the Earth’s rotation around its axis and the seasonal rhythm caused by the Earth’s rotation around the sun. Therefore, Bünning^16^ proposed the functional involvement of the circadian clock in seasonal time measurement. In line with this proposition, the involvement of genes of the circadian clock in photoperiodism was verified in several insect species (e.g.^17–20^.). In other cases, it has been more controversially discussed whether the circadian clock perceives photoperiod^21,22^. Emerson et al*.*^23^ found that the circadian clock and the photoperiodic clock that controls diapause can evolve independently, and there is an ongoing debate as to if and/or to what extent the circadian clock and the timer of photoperiod have the same underlying genetic mechanism^22,24^. In the case of *Daphnia*, Roulin et al*.*^25^ have demonstrated in a QTL study that a variation in a rhodopsin photoreceptor gene plays a significant role in the variation of timing of resting stage induction that is not part of the circadian clock.

It is not known whether the expression of the circadian clock genes persists during diapause in *Daphnia*. It is well imaginable that the counting of the number of elapsed clock cycles contributes to the timing of diapause termination; in addition, ephippia often rest in sediments that are not reached by light. However, a light stimulus is needed to initiate development in resting eggs. *Daphnia* diapause is most effectively terminated by blue and UV-light stimuli^26^. Interestingly, *cryptochrome 2* (*cry2*) is a gene in *Daphnia*’s putative circadian clock system^27^ that has been shown to be expressed in a cyclic manner over a 24-h day-night cycle^28,29^. In other organisms (e.g. spider mites^30^) the photoperiodic clock necessary for termination of diapause is probably not identical to the circadian clock. However, the involvement of *cry 2* in diapause termination of *Daphnia* is likely: In a QTL study, Czypionka et al.^31^ have identified three isoforms of an ELKS/Rab6 interacting/Cast 396 family member protein (ERC; homologous to the gene *bruchpilot* (*brp*) in insects) to be potentially involved in diapause termination. Interestingly, *brp* interacts with the circadian clock although it is not part of the core circadian system^32^. Analogous to the light-dependent degradation of the circadian clock gene *timeless* (*tim*), it is degraded by cryptochrome^33^. Therefore, the circadian clock might play a significant role in diapause termination in *Daphnia*.

We hypothesize that the core genes of *Daphnia*’s circadian clock in ephippia are expressed differently in the initiation, resting, and termination phases of diapause. We further hypothesize that *cry 2* and *brp* are highly expressed in ephippia either during initiation or termination of diapause in order to provide enough mRNA and/or photoreceptor molecules for immediate diapause termination and thus for a quick resumption of development of resting eggs. Therefore, we measured expression of *brp* and five core clock genes (*cry 2*, *tim*, *period* (*per*), *clock* (*clk*) and *cycle* (*cyc*)) that had previously been demonstrated to show a day-time dependent expression in *D. pulex*^28^. Gene expression of these genes was measured in sexually produced embryos of *D. magna* that are destined to go into a phase of suspended animation^34^. We selected developmental stages based on cell count in which diapause is initiated, maintained and terminated. Asexually produced embryos develop continuously, and also here we measured gene expression in the comparative developmental stages. This allowed us to determine clock gene expression during continuous development and development intermitted by a phase of suspended animation.

## Material and methods

### Culture conditions

We raised a population of the *D. magna* clone ‘Elias’ from Mount Sinai, Egypt, and a *D. magna* clone FT442 from Finland (kindly provided by Dieter Ebert) as published in^34^: All animals of the culture and the experiments were raised in 1 L glass jars (WECK, Germany) filled with ADaM medium^35^) in temperature-controlled incubators at 20 °C ± 0.1 °C and under different light conditions (for asexually produced embryos: 16:8 day:night; for sexually produced embryos: 8:16 day:night). Animals were fed the algae *Acutodesmus obliquus *ad libitum, > 1.5 mg C/L. To ensure clonal reproduction, females were kept at low densities (about 30 adult females per 800 mL of ADaM in a 1 L jar, Weck; Germany). Sexual reproduction was induced by maintaining the clones under shortened photoperiodic conditions (8 h: 16 h light: dark cycle) at 20 °C ± 0.1 °C, with low food levels and via crowding. To create the crowded conditions, we cultured more than 50 adult male and female animals in 800 mL ADaM under conditions of limited food supply, < 1 g C/L. Food concentration was determined by measuring the algae’s optical density (at 800 nm). Carbon content was adjusted to a standard curve available in the lab.

We collected asexually produced embryos from clone ‘FT442’ and sexually produced clones were crosses of ‘FT442′ females and ‘Elias’ males.

### Ovulation monitoring of sexually and asexually reproducing *D. magna*

We monitored sexually and asexually reproducing females (as described in^34^; Table 1) in order to collect timely staged sexually and asexually produced *Daphnia* embryos. Sexually and asexual produced embryos can already be distinguished within the ovary (for details see^34^). Upon the appearance of either type, each female was individually transferred into a 50 mL snap cap vial filled with 40 mL ADaM and fed the algae *Acutodesmus obliquus *ad libitum. Asexual females were in their vials, while one male of a different clone was transferred to the sexually reproducing female. We then checked all females at 15 min intervals to determine the time point of ovulation. From this time point onwards, we collected time-dependent stages of asexually and sexually produced embryos (listed in Table 1) during the light phase of the respective photoperiod 8 h:16 h light: dark cycle (sexually produced embryos) and 16 h:8 h light: dark cycle (asexually produced embryos) and during daytime from 10 a.m. to 4 p.m. (Central European Standard Time). Sexually produced embryos that had entered diapause were transferred to dark and cold conditions; these conditions are necessary to prevent hatching. Resurrection was initiated by exposure to daylight (Osram Biolux L, 30 W/965) in an acclimatized room at 20° C ± 0.1 °C (in a long photoperiod with a 16:8 light: dark cycle), and respective stages were again collected during the daytime from 10 a.m. to 4 p.m. (Table 1). Target stages were selected with respect to the developmental time point based on cell count or explicit morphological features as published in^34^. All work was performed at 20 °C ± 0.1 °C to ensure timely correlated development of biological replicates.

**Table 1:** Comparative sampling stages based on cell number of sexually and asexually produced embryos collected for qPCR.

Stage	Time of collection in sexually produced embryos	Time of collection in asexually produced embryos
1000 cell stage	< 24 h-mitotic active stage	10 h-mitotically active stage
3500 cell stage	48 h-deceleration stage, pre-diapause	15 h-mitotically active stage
3500 cell stage	74 h-stationary phase	–
3500 cell stage	1 month in diapause	–
3500 cell stage	11 months in diapause	–
Unknown	1 d reactivation	–
Unknown	5 d reactivation	–
Unknown	12 d reactivation	–
Unknown	19 d reactivation	–
> 7000 cells	Revived 1-appearance of the 2nd antennae	24 h- mitotically active stage; appearance of the 2nd antennae
> 7000 cell stage	Revived 2-appearance of bright red eye spots	Appearance of bright red eye spots
	Revived 3-eye spots fused and black	Eye spots fused and black

### Fixation of sampling stages

When the animals reached the respective developmental stage, they were flash-frozen in liquid nitrogen and stored at − 80 °C until RNA extraction was conducted. When sexually produced embryos were encapsulated in an ephippium, they were dissected using a fine forceps and flash frozen without the ephippium. We collected three timely correlated biological replicates consisting of 30 embryos (stages with < 3500 cells) and 15 embryos (stages with > 3500 cells).

### RNA extraction

Tissue samples were thawed on ice, homogenized using a pistil and then extracted with the ReliaPrep RNA Miniprep system (Promega, Germany) for tissues as according to the manufacturer’s instructions. The RNA was quality checked; only samples with A_260/280_ ~ 2.0 and A_260/230_ ~ 2.0–2.2 were used. The RNA integrity index was determined with the help of an Experion microchip reader (Biorad, Germany) and a StdSens RNA kit. Only samples with a RIN > 8.0 were taken for qPCR. RNA quantity was determined with a Qubit RNA broad range assay kit (Thermo Fisher Scientific, Germany).

### One step RT-qPCR

Reverse transcription quantitative PCR (RT-qPCR) was performed using the Luna Universal One Step RT-qPCR kit (New England Biolabs, Germany) as according to the manufacturer’s protocol. In total, 10 ng RNA was added to a total reaction volume of 10 µl so that 1 ng RNA was reverse transcribed and amplified with specific primers. Primer pairs (Table 2) were added at a concentration of 0.4 µM, and amplification was performed at 60 °C with 40 cycles. All reactions were finalized by a melting curve step, giving constant melting peaks but in the non-template and the non-reverse transcription controls. Plates were set up in technical duplicates. Due to the number of samples, replicates and genes, multiple plates were used. These plates were controlled for comparative results by adding a two standard RNA samples that were run with the reference gene primer *tbp*^36^. Differences between these Cqs of both RNAs of all the individual plates were lower than 0.2%.

**Table 2:** qPCR primers for *Daphnia magna* clock genes.

Gene	Abbreviation	Primer forward (5′-3′)	Primer reverse (5′-3′)	T_melt_	Amplicon size (bp)	Gene origin
Clock	*clk*	tccttttgaagttctcgggaca	gcttcatgacaggtagaaactttc	60 °C	80	scaffold00547
Cycle	*cyc*	ttttattcgtcgtgggctgc	aataattgagcacttgagacaccg	60 °C	75	scaffold03242
Cryptochrome 2	*cry2*	tgctactagacgcagattggtc	actttcctgccaaatctgacag	60 °C	115	scaffold00687
Timeless	*tim*	tccgcatcattggctacact	cgatggctgtgattactgatgc	60 °C	111	scaffold03376
Period	*per*	cggccggaattcaacagatg	tgctcggcttccatttctgt	60 °C	117	scaffold02670
Bruchpilot	*brp*	cacaacgatggcgttcacgtatt	gtcttctcagccacttctgacgt	56 °C	149	Dm_Bassoon
Tata-box binding protein	*tbp*	gcagggaagtttagtttctgga	tggtatgcacaggagcaaag	60 °C	88	Heckmann et al*.* 2006

Listed are gene names, abbreviations, primer sequences, melting temperature (T_melt_), amplicon sizes and the origin of *D. magna* sequences for which *D. pulex* sequences (for gene IDs see Schwarzenberger & Wacker 2015) were blasted against the *D. magna* genome v.2.4 (wfleabase.org). Delineated are the scaffolds on which the blast hits were positioned. Tata-box binding protein (Heckmann et al*.* 2006) was used as reference based on result obtained from RefFinder^38^.

### Data analysis

Primer efficiency was determined using LinReg^36^. The reference gene *tbp*^37^ was validated using RefFinder^38^ and found to be stably expressed over all tested stages. We had to rely on this single reference gene only, as other standard reference genes (i.e. *STX16, actin WARS, 18S*) were strongly regulated between developmental stages and between sexually and asexually produced embryos. Unfortunately, *tbp* expression was not stable between sexually and asexually produced embryos, a fact which prevented us from directly comparing gene expression between the embryo types. Differential gene expression between all tested stages was analyzed as according to the Pfaffl method in REST^39^. The mathematical model used is based on the correction for exact PCR efficiencies and the mean crossing point deviation between sample group(s) and control group(s). Subsequently, the expression ratio results of the investigated transcripts are tested for significance by a Pair Wise Fixed Reallocation Randomisation Test.

## Results

**Figure 1:**
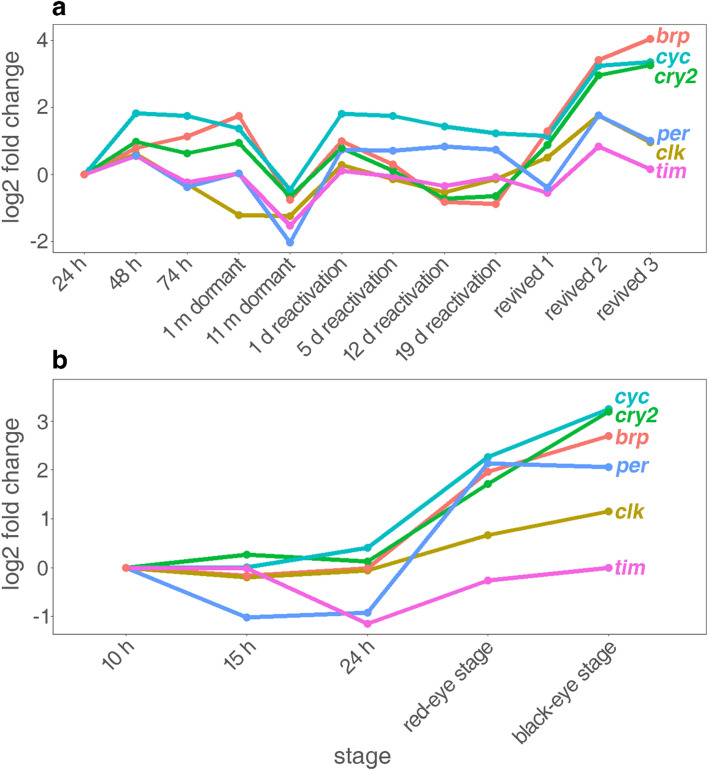
mRNA expression of all six genes across developmental stages in (**a**) sexually and (**b**) asexually produced *D. magna* embryos. Displayed is the log2 fold change of each stage relative to the first stage, i.e. 24 h in sexually produced embryos and 10 h in asexually produced embryos. For more details see supplemented heatmaps (Supplementary 1).

We analyzed log2 fold changes in gene expression of circadian clock genes and a putatively associated gene during the development of sexually produced embryos that are about to go into diapause. Moreover, we resurrected these embryos and screened for gene expression changes (Fig. 1a). In order to see diapause-associated changes in circadian clock gene expression, we compared these gene expression patterns to gene expression patterns in asexually produced embryos (Fig. 1b). More detailed information on gene expression differences between all stages and *p*-values can be found in the supplemented heatmaps (Supplementary 1).

### Gene expression patterns during diapause preparation in sexually produced embryos

In the preparation phase of diapause at 48 h post ovulation (Fig. 1a), *clk* and *cyc* mRNA is significantly upregulated in comparison to 24 h post ovulation. In comparison, *tim* shows a significant weaker log2-fold expression change when 48 h sexual embryos are compared with 24 h embryos, whereas *per* gene expression was not changed. *Cry2* mRNA is significantly upregulated 48 h post ovulation in comparison to 24 h*. Brp* shows a tendency of being upregulated in this developmental stage*.*

### Gene expression patterns during diapause of sexually produced embryos

In stages when morphological development has come to a halt, i.e. at 74 h and 1-month dormant (Fig. 1a), *clk*, *per* and *tim* expression does not change significantly in comparison to the 24 h stage, but was downregulated in comparison to the 48 h stage (except *per*). *Clk*, *per* and *tim* expression is significantly downregulated in 11-month dormant embryos compared with all previous developmental stages. *Cyc* expression remains at a stable expression level until 1-month domant. In 11-months dormant embryos, *cyc* expression is not significantly different from *cyc* expression in 24 h embryos, but is significantly reduced in comparison to the other previous developmental stages. In comparison to 24 h, *cry2* expression is significantly upregulated in all stages until 1-month dormant, but is significantly downregulated in 11-months dormant embryos. In 1-month dormant embryos, *brp* expression is significantly increased in comparison to the previous developmental stages, but is significantly downregulated in 11-months dormant embryos.

### Gene expression patterns during resurrection of sexually produced embryos

Upon resurrection through light exposure (Fig. 1a), *cyc*, *per* and *tim* gene expression is significantly increased in reactivated embryos in comparison to 11-months dormant embryos and either reached similar (*tim*) or significantly higher gene expression levels than before diapause (cyc: 1 and 19 d reactivation; per: 1 to 19 d reactivation). *Clk, cry2* and *brp* gene expression is also significantly increased in reactivated embryos in comparison to 11-months dormant embryos. With ongoing reactivation, gene expression levels are lower than in pre-diapause stages (*clk*: 12 d reactivation; *cry2*: 12 to 19 d reactivation; *brp*: 19 d reactivation).

### Gene expression patterns in resurrected and developing sexually produced embryos

Active development in sexually produced embryos was determined based on the appearance of morphological features, i.e. the second antennae (revived 1), red eye stage (revived 2) and black eye stage (revived 3; Table 1). Gene expression of *clk*, *cry2* and *brp* increases significantly after reactivation to similar (*clk*, *brp*) or higher levels (*cry2*) than before diapause. *Cyc* gene expression first decreases to similar levels as before diapause (revived 1) and then significantly increases in gene expression (revived 2 and 3). *Per* and *tim* gene expression decreases after resurrection (revived 1), then increase in gene expression (revived 2), before returning to similar levels as before diapause (revived 3).

### Gene expression patterns across development in asexually produced embryos

In asexually produced embryos, *clk* and *cyc, brp, cry2* become significantly upregulated in the red- and black-eye stages (Fig. 1b), whereas *tim* is stably expressed across all developmental stages except in the 24 h post ovulation stage when gene expression is significantly reduced.

## Discussion

*Daphnia*’s core clock shows a 24-h pattern of gene expression in response to changes in day and night^28,29^. *Daphnia* can also adjust its clock gene expression to different photoperiods (i.e. one clone of *D. pulex* shows higher and longer *per* gene expression during longer nights; Schwarzenberger, A. & Wacker, A. unpublished data). This suggests that the clock is not only circadian but also that it measures photoperiod. Therefore, it is reasonable to assume that *Daphnia*’s core clock genes act not only as a clock (a preceptor of day-length), but also as a counter of shortening days in order to induce diapause.

We found that induction of diapause in *Daphnia magna* involves a general expression increase of the core clock genes and the clock-associated gene *brp*. We have recently elucidated that at the point in time when the embryos are still in the mother’s brood pouch, mitotic activity decelerates and comes to a halt 50 h post ovulation^34^. In line with this, we found that the up-regulation of *tim*, *per* and *clk* is completed after 48 h post ovulation, and we have previously found and again find here that diapause is prepared until 48 h post ovulation. The halt of mitotic activity might be caused by an arrest of the circadian clock. Since *clk* is no longer expressed, translated clk and cyc can no longer form into a hetero-dimer. This hetero-dimer is necessary as a transcription factor binding to the E-box of *per* and *tim*^40^, and so gene expression is also downregulated. The circadian clock probably stops without *per* and *tim* transcription.

In the cases of *cyc*, *cry2* and *brp*, increased gene expression lasts until 74 h post ovulation. At this stage, development is completely suspended and embryos are encapsulated in ephippia which are shed during the mother’s next molting cycle^34^. Since expression of these genes is still stably increased even at developmental arrest and until one month of diapause (or in case of *brp* is even further increased), this allocation of additional mRNA molecules might allow continuous mRNA translation into proteins also during developmental arrest. By this, receptor molecules of e.g. *cry2* can be continuously synthesized to be functional as a blue light sensor that may enable diapause termination upon stimulation. Similarly, since *cry2* and *brp* are connected (i.e. brp is degraded by cry2 after light stimulation^33^), a provision of *brp* mRNA or translated protein at the time point of diapause termination is then necessary. We therefore anticipate that *cry2* is another photoreceptor gene (besides a rhodopsin gene^25^) that plays a significant role in the variation of timing of resting-stage induction in *Daphnia*. Interestingly, *cry2* seems to be involved in diapause in insects as well: In *Drosophila*, allelic differences in *cry2* (and *tim*) were associated with differences in the incidence of diapause^41^. In the case of *cyc*, a continuous provision of mRNA or translated proteins might be necessary to prepare the restart of the circadian clock immediately after diapause termination. If—at the time point of diapause termination—the gene expression of *clk* is initiated, the formation of the clk-cyc hetero-dimer is possible in a short amount of time.

During deep diapause (i.e. 11 months post ovulation), expression of all clock genes is down-regulated. This is in line with findings for *per* and *cry* expression in diapausing adult females of an insect (*Pyrrhocoris apterus*^42^). Therefore, sustaining *Daphnia*’s clock gene expression is not necessary for active maintenance of diapause and is arrested similarly to other genes involved in growth, development and metabolism. Furthermore, the clock does not act as a counter of days until diapause termination, because the daily rhythmicity is probably arrested during diapause. At the time point of reactivation with day-light, all core clock genes and the associated gene *brp* increase in expression levels. This has also been observed in adults of the insect *Pyrrhocoris apterus*, for which *per* and *cry* increase after diapause termination^42^. However, in the pupae of another insect species, *Rhagoletis pomonella*, no change in clock gene expression has been found between early and late diapause and diapause termination^43^. In *Daphnia*, all genes show a strong increase in gene expression in comparison to deep diapause which levels out (or slightly decreases) during all stages of reactivation (where there are no signs of morphological differentiation). This level probably represents the onset of the daily cycling of the clock which is necessary for metabolism and other physiological responses during reactivation.

Both revived and asexually produced embryos grew in the same photoperiod and developed into parthenogenetic females. Therefore, it is not surprising that gene expression of the clock is similar during development from embryo to the black-eye stage. In both cases, gene expression increases continuously over developmental progression, or—in case of *tim*—gene expression first decreases and then increases both in sexual and asexual embryos. A higher clock gene expression is probably necessary in order to maintain the increasing circadian metabolic activity of growing embryos.

To our knowledge, this is the first report describing the expression of the genes of the core clock of embryos of a crustacean over a whole diapause cycle (i.e. before, during and after diapause). We found that the clock is differentially expressed during diapause induction but not during its maintenance; furthermore, the photoreceptor *cry2* and the downstream *brp* are highly expressed in the late induction and early diapause phase, probably in order to store mRNA or molecules necessary for immediate diapause termination due to a light stimulus. After reactivation, both sexually and asexually produced embryos show a similar pattern of gene expression during development to parthenogenetic females.

Diapause is an essential phase during the life cycle of many arthropods; survival is not possible in deleterious living conditions without the sexual production of resting stages. Our study is a crucial addition to the understanding of the molecular basis of diapause induction, maintenance and termination. Furthermore, based on our findings, RNAi (reverse genetics) knock-down of certain clock genes is the next logical step to test whether diapause or its termination is still possible with reduced gene expression in vivo.

## Supplementary information

Supplementary Information

## Data Availability

Data are provided in the appendix.
