# Supplementary material for: The expression of circadian clock genes in Daphnia magna diapause
Source: Sci Rep. 2020 Nov 16;10:19928. doi: 10.1038/s41598-020-77065-3 (PMC7669902; doi:10.1038/s41598-020-77065-3)
Supplement: Supplementary file 1 — Supplementary Information [file 41598_2020_77065_MOESM1_ESM.docx]

## The expression of circadian clock genes in *Daphnia magna* diapause

Anke Schwarzenberger, Luxi Chen & Linda C. Weiss

**Extended Results**

We analyzed log2 fold changes in gene expression of circadian clock genes and a putatively associated gene during the development of sexually produced embryos that are about to go into diapause. Moreover, we resurrected these embryos and screened for gene expression changes. In order to see diapause-associated changes in circadian clock gene expression, we compared these gene expression patterns to gene expression patterns in asexually produced embryos.

**Gene expression patterns during diapause preparation in sexually produced embryos**

In the preparation phase of diapause at 48 h post ovulation, *clk* and *cyc* mRNA is significantly upregulated in comparison to 24 h post ovulation by 0.61-log2-fold (*clk)* and 1.83-log2-fold (*cyc)* respectively (Fig. S1 a/b). In comparison, *tim* and *per* show a weaker log2-fold expression change of 0.54 (*P*<0.05) and 0.56 (not statistically supported) when 48 h sexual embryos are compared with 24 h embryos (Fig. S2 a/b). *Cry2* mRNA is significantly upregulated by 0.97-log2-fold *P<0.001* 48 h post ovulation in comparison to 24 h (Fig. S3 a)*. Brp* shows a tendency of being upregulated in this developmental stage by 0.78-log2-fold *(P*=0.06; Fig. S4 a)*.*

**Gene expression patterns during diapause of sexually produced embryos**

In stages when morphological development has come to a halt, i.e. at 74 h, *clk* expression is downregulated (-0.89-log2-fold, *P*<0.05) in comparison to the 48 h stage, for which gene expression was significantly increased in comparison to the 24 h stage (Fig. S1 a). Therefore, this is reflected in the comparison of the 74 h with the 24 h stage where gene expression does not change significantly (-0.28-log2-fold, *P*>0.05). *Clk* expression is stabilized when comparing 1‑month dormant stages with 74 h embryo stages (-0.93-log2-fold, *P*>0.05), and significantly downregulated in 11-month dormant embryos compared with all previous developmental stages (24 h: -1.24, *P*<0.001; 48 h: -1.85, *P*<0.001, 74 h: -0.96, *P*<0.001). In comparison to 1‑month dormant embryos, gene expression is stable in 11‑month dormant embryos (-0.03-log2-fold, *P*>0.05).

*Cyc* expression remains at a stable expression level, so that when comparing 74 h to 48 h (-0.08-log2-fold, *P*>0.05) and 1-month dormant with 74 h (-0.39-log2-fold), gene expression is stable. However, expression increased 1.75-log2-fold (*P*<0.05) when comparing 74 h with 24 h and 1.36-log2-fold (*P*<0.05) comparing 1-month dormant with 24 h post ovulation (Fig. S1 b). In 11-months dormant embryos, *cyc* expression is not significantly different from *cyc* expression in 24 h embryos (-0.46-log2-fold, *P*>0.05), but is significantly reduced in comparison to 48 h (-2.29-log2-fold, P<0.001), 74 h (-2.21-log2-fold. P<0.001) and 1-month dormant (-1.82-log2-fold, *P<0.001*).

*Tim* expression is significantly downregulated when comparing 74 h (-0.78-log2-fold, *P*<0.001) with 48 h, and stays at this expression level when comparing 1-month dormant with 74 h (0.26-log2-fold, P>0.05; Fig. S2 a). One month dormant embryos show no differential expression of *tim* mRNA in comparison to the developing stages i.e. 48 h (-0.52-log2-fold, *P*=0.067) and 24 h (0.03-log2-fold; *P*>0.05). In 11 months dormant embryos, *tim* expression is significantly downregulated in comparison to all previous stages (i.e. 24 h: -1.53-log2-fold, *P*<0.001, 48 h: -2.08-log2-fold, *P*<0.001, 74 h: -1.30-log2-fold, *P=*0.051, 1 month dormant: -1.56-log2-fold, *P*<0.001).

*Per* expression is significantly downregulated when comparing 74 h (-0.95-log2-fold, *P*<0.001) with 48 h and this expression level is slightly increased when comparing 1-month dormant with 74 h (0.41-log2-fold, P<0.05; Fig. S2 b). One-month dormant embryos show no differential expression of *tim* mRNA in comparison to the developing stages, i.e. 48 h (-0.54-log2-fold, *P*=0.067) and 24 h (0.03-log2-fold; *P*>0.05). In eleven-months dormant embryos, *per* expression is significantly downregulated in comparison to all previous stages (i.e. 24 h: -2.04-log2-fold, *P*<0.05, 48 h: -2.60-log2-fold, *P*<0.001, 74 h: -1.65-log2-fold, *P<0-001*, 1‑month dormant: -2.06-log2-fold, *P*<0.01).

*Cry2* expression is significantly upregulated when 74 h is compared with 24 h (0.62-log2-fold, *P*<0.05) but *cry2* mRNA is downregulated (-0.35-log2-fold, P<0.001; Fig. S3 a) in comparison to 48 h post ovulation. In 1-month dormant embryos, *cry2* expression is still increased in comparison to the 24 h post ovulation stage (0.94-log2-fold, *P*<0.05), but this expression stabilizes when comparing 1-month dormant embryos with the 48 h (-0.03-log2-fold, P>0.05) and the 74 h post ovulation stages (0.31-log2-fold, P>0.05). In eleven-months dormant embryos, *cry2* expression is significantly downregulated in comparison to all previous stages (i.e. 24 h: -0.66-log2-fold, *P*<0.001, 48 h: -1.63-log2-fold, *P*<0.001, 74 h: -1.28-log2-fold, tendency of *P=*0.051, 1-month dormant: -1.60-log2-fold, *P*<0.05).

*Brp* expression shows a tendency towards upregulation when 74 h is compared with 24 h (1.14-log2-fold, *P*=0.06), but *brp* mRNA expression is stable (0.36-log2-fold, P>0.05; Fig. S4 a) in comparison to 48 h post ovulation. In 1-month dormant embryos, *brp* expression is still increased in comparison to the 24 h post ovulation stage (1.75-log2-fold, *P*<0.05), 48 h (0.97-log2-fold, P<0.05) and 74 h post ovulation stage (0.61-log2-fold, P<0.05). In eleven-months dormant embryos, *brp* expression is significantly downregulated in comparison to all previous stages (i.e. 24 h: -0.76-log2-fold, *P*<0.01, 48 h: -1.54-log2-fold, *P*<0.05, 74 h: -1.89-log2-fold, tendency of *P=*0.07, 1-month dormant: -2.51-log2-fold, *P*<0.05).

**Gene expression patterns during resurrection of sexually produced embryos**

Upon resurrection through light exposure, c*lk* gene expression is significantly increased in reactivated embryos in comparison to 11-months dormant embryos (1 d reactivation: 1.52-log2-fold, *P*<0.001; 5 d reactivation: 1.09-log2-fold, *P*<0.001; 12 d reactivation 0.70-log2-fold, *P*<0.001; but not significantly increased after 19 d reactivation: 1.10-log2-fold, *P*>0.05; Fig. S1 a). Also in comparison to 1-month dormant embryos, expression in 1-day reactivated embryos is increased 1.49-log2-fold, albeit not statistically supported (*P*>0.05). Gene expression in 1-day reactivated embryos is the same or even lower than gene expression prior to diapause (i.e. 24 h: 0.28-log2-fold, *P=*0.096, 48 h: -0.33-log2-fold, *P*<0.05). With ongoing reactivation, the gene expression level is the same or is lower than in pre-diapause stages (24 h vs. 5 d reactivation: -0.15-log2-fold, *P>*0.05 , 24 h vs. 12 d reactivation: -0.54-log2-fold, *P*<0.05, 24 h vs. 19 d reactivation -0.14-log2-fold, *P*>0.05).

*Cyc* gene expression is significantly increased in reactivated embryos in comparison to 11‑months dormant embryos (1 d reactivation: 2.26-log2-fold, *P*<0.001; 5 d reactivation: 2.20-log2-fold, *P*<0.05; 12 d reactivation 1.89-log2-fold, *P*<0.001; 19 d reactivation: 1.69-log2-fold, *P<*0.001; Fig. S1 b). Also in comparison to 1-month dormant embryos, expression in 1-day reactivated embryos shows a tendency to be increased 0.44-log2-fold (*P*=0.059). Gene expression in 1-day reactivated embryos is on the same level with or is even higher than gene expression prior to diapause (i.e. 24 h: 1.80-log2-fold, *P<*0.05, 48 h: -0.03-log2-fold, *P*>0.05). With ongoing reactivation, gene expression is on the same level with or is higher than pre-diapause stages (24 h vs. 5 d reactivation: 1.74-log2-fold, *P=0.061* , 24 h vs. 12 d reactivation: 1.43-log2-fold, *P*=0.062, 24 h vs. 19 d reactivation 1.23-log2-fold, *P*>0.05).

*Tim* gene expression is significantly increased in reactivated embryos in comparison to 11‑months dormant embryos (1 d reactivation: 1.64-log2-fold, *P*<0.001; 5 d reactivation: 1.47-log2-fold, *P=*0.059; 12 d reactivation 1.18-log2-fold, *P*<0.001; 19 d reactivation: 1.45-log2-fold, *P<*0.001; Fig S2 a). In comparison to 1-month dormant embryos, expression in 1‑day reactivated embryos is not significantly different (0.08-log2-fold). Gene expression in 1‑day reactivated embryos is at the same level with or is even lower than gene expression prior to diapause (i.e. 24 h: 0.10-log2-fold, *P>*0.05, 48 h: -0.44-log2-fold, *P<*0.001). With ongoing reactivation, gene expression is at the same level as pre-diapause stages (24 h vs. 5 d reactivation: -0.06-log2-fold, *P>0.05* , 24 h vs. 12 d reactivation: -0.35-log2-fold, *P>*0.05 24 h vs. 19 d reactivation -0.09-log2-fold, *P>*0.05).

*Per* gene expression is significantly increased in reactivated embryos in comparison to 11‑months dormant embryos (1 d reactivation: 2.78-log2-fold, *P*<0.05; 5 d reactivation: 2.74-log2-fold, *P*<0.05; 12 d reactivation 2.86-log2-fold, *P*<0.001; 19 d reactivation: 2.77-log2-fold, *P<*0.001; Fig. S2 b). Also in comparison to 1-month dormant embryos, expression in 1‑day reactivated embryos shows a tendency to be increased by 0.71-log2-fold (*P*=0.08). Gene expression in 1-day reactivated embryos levels with or is even higher than gene expression prior to diapause (i.e. 24 h: 0.74-log2-fold, *P<*0.05, 48 h: 0.17-log2-fold, *P>*0.05). With ongoing reactivation, gene expression is at the same level as or is higher than pre-diapause stages (24 h vs. 5 d reactivation: 0.70-log2-fold, *P<0.05* , 24 h vs. 12 d reactivation: 0.83-log2-fold, *P*<0.001, 24 h vs. 19 d reactivation 0.74-log2-fold, *P<*0.05).

*Cry2* gene expression is significantly increased (1 d reactivation: 1.44-log2-fold, *P*<0.001) or at the same level (5 d reactivation: 0.76-log2-fold, *P>0.05*; 12 d reactivation -0.06-log2-fold, *P>*0.05; 19 d reactivation: 0.02-log2-fold, *P>*0.05, Fig. S3 a) in reactivated embryos in comparison to 11-months dormant embryos. Expression of 1-day reactivated embryos is not significantly different from that of 1-month dormant embryos (-0.15-log2-fold, *P>*0.05). Gene expression in 1‑day reactivated embryos is significantly increased or level in comparison to stages prior to diapause (i.e. 24 h: 0.78-log2-fold, *P<*0.001, 48 h: -0.19-log2-fold, *P>*0.05). With ongoing reactivation, gene expression levels are the same or lower than in pre-diapause stages (24 h vs. 5 d reactivation: 0.10-log2-fold, *P>0.05* , 24 h vs. 12 d reactivation: -0.72-log2-fold, *P<*0.001 24 h vs. 19 d reactivation -0.64-log2-fold, *P<*0.001).

*Brp* gene expression shows a slight but not significant increase in reactivated embryos in comparison to 11‑months dormant embryos (1 d reactivation: 1.75-log2-fold, *P*=0.061; 5 d reactivation: 1.06-log2-fold, *P=0.07*; 12 d reactivation -0.06-log2-fold, *P>*0.05; 19 d reactivation: -0.13-log2-fold, *P>*0.05; Fig. S4 a). In comparison to 1-month dormant embryos, expression in 1-day reactivated embryos is significantly decreased by -0.76-log2-fold, *P<*0.001. Gene expression in 1-day reactivated embryos is at the same level as gene expression in stages prior to diapause (i.e. 24 h: 0.99-log2-fold, *P*=0.079, 48 h: 0.21-log2-fold, *P>*0.05). With ongoing reactivation, gene expression decreases in comparison to the pre-diapause stage (24 h vs. 5 d reactivation: 0.30-log2-fold, *P>0.05*, 24 h vs. 12 d reactivation: -0.82-log2-fold, *P>*0.05, 24 h vs. 19 d reactivation -0.89-log2-fold, *P<*0.001).

**Gene expression patterns in resurrected and developing sexually produced embryos**

Active development in sexually produced embryos was determined based on the appearance of morphological features, i.e. the second antennae (revived 1), red eye stage (revived 2) and black eye stage (revived 3; Tab. 1). Gene expression of *clk* shows a strong tendency to remain at an increased level in comparison to the 11-month dormant stage (revived 1: 1.74-log2-fold, *P=0.069*, revived 2: 3.01-log2-fold, *P=0.063*, revived 3: 2.20-log2-fold, *P<0.05*; Fig. S1 a). In comparison to the pre-diapause stage, 24 h post ovulation gene expression shows a slight but not significant increase in all three developmental stages (revived 1: 0.50-log2-fold, *P=0.061*, revived 2: 1.77-log2-fold, *P=0.07*, revived 3: 0.96-log2-fold, *P=0.065*).

Gene expression of *cyc* remains at an increased level in comparison to the 11-month dormant stage (revived 1: 1.61-log2-fold, *P<0.05*, revived 2: 3.70-log2-fold, *P<0.05*, revived 3: 3.82-log2-fold, *P<0.05*; Fig. S1 b)*.* In comparison to the pre-diapause stage, 24 h post ovulation gene expression shows a slight but not significant or even significant increase in all three developmental stages (revived 1: 1.15-log2-fold, *P=0.095*, revived 2: 3.24-log2-fold, *P=0.06*, revived 3: 3.36-log2-fold, *P<0.05*).

Gene expression of *tim* remains at an increased level in comparison to the 11-month dormant stage (revived 1: 0.99-log2-fold, *P<0.05*, revived 2: 2.37-log2-fold, *P<0.05*, revived 3: 1.69-log2-fold, *P<0.001*; Fig. S2 a)*.* In comparison to the pre-diapause stage, 24 h post ovulation gene expression shows a level expression or significant decrease (revived 1: -0.55-log2-fold, *P<0.001*, revived 2: 0.84-log2-fold, *P<0.05*, revived 3: 0.16-log2-fold, *P>0.05*).

Gene expression of *per* stays at higher level than the 11-month dormant stage (revived 1: 1.65-log2-fold, *P<0.05*, revived 2: 3.80-log2-fold, *P<0.001*, revived 3: 3.04-log2-fold, *P<0.01*; Fig. S2 b). In comparison to the pre-diapause stage, 24 h post ovulation gene expression shows a level expression or significant increase (revived 1: -0.39-log2-fold, *P>0.05*, revived 2: 1.77-log2-fold, *P<0.001*, revived 3: 1.00-log2-fold, *P=0.069*).

Gene expression of *cry2* stays at higher level than the the 11-month dormant stage (revived 1: 1.54-log2-fold, *P<0.05*, revived 2: 3.61-log2-fold, *P<0.001*, revived 3: 3.91-log2-fold, *P<0.05*; Fig. S3 a). In comparison to the pre-diapause stage, 24 h post ovulation gene expression shows significant increase (revived 1: 0.88-log2-fold, *P<0.05*, revived 2: 2.95-log2-fold, *P<0.001*, revived 3: 3.25-log2-fold, *P<0.001*).

Gene expression of *brp* remains at a higher level than the 11-month dormant stage (revived 1: 2.04-log2-fold, *P=0.056*, revived 2: 4.17-log2-fold, *P<0.05*, revived 3: 4.80-log2-fold, *P<0.05*; Fig. S4 a). In comparison to the pre-diapause, stage 24 h post ovulation gene expression shows slight but not significant increase (revived 1: 1.28-log2-fold, *P=0.072*, revived 2: 3.41-log2-fold, *P=0.07*, revived 3: 4.04-log2-fold, *P=0.074*).

**Gene expression patterns across development in asexually produced embryos**

In asexually produced embryos, *clk* and *cyc, per, brp, cry2* become significantly upregulated during development (Fig. S1 c/d, S2 d, S3 b, S4 b). Only *tim* is stably expressed across all developmental stages (Fig. S2 c). Gene expression is significantly reduced in the 24 h post ovulation stage in comparison to the 10 h post ovulation.


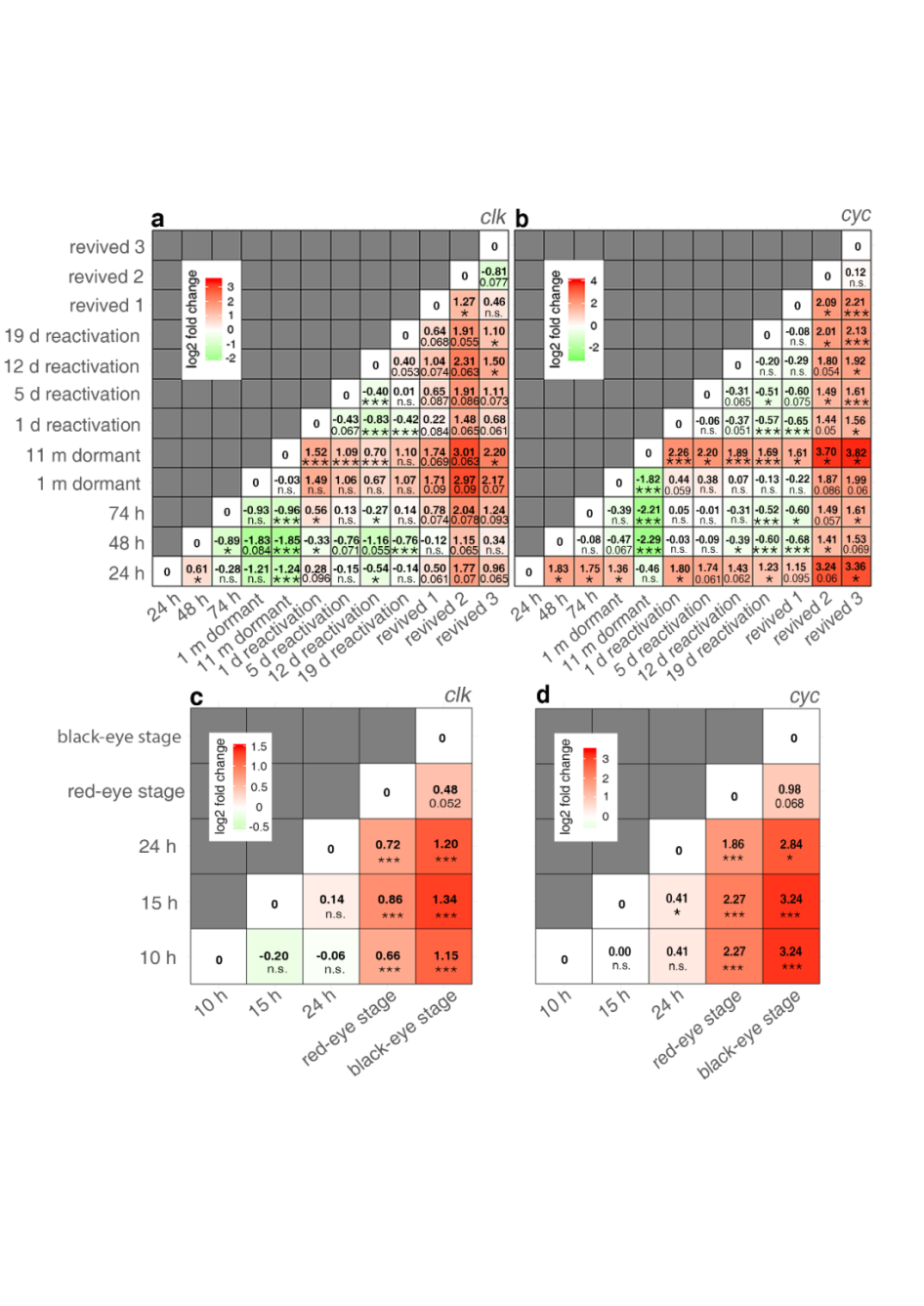


**Fig. S1:** Heatmaps (generated with the packages “ggplot2” and “reshape2” in R Version 1.1.383 [1,2,3], displaying the log2 fold changes of gene expression between all tested stages and the statistical results obtained from REST analysis) of *clk* and *cyc* mRNA expression patterns across developmental stages in sexually and asexually produced *D. magna* embryos. Displayed are the log2 fold expression change (top value) and significance level (bottom indicated significance level * *P*<0.05; ** *P*<0.01; *** *P*<0.001; n.s.= not significant when *P >0.1*) in the developmental stage depicted on the x-axis with respect to the stage on the y-axis. **a:** *Clk* gene expression log2 fold changes and significance levels in sexually produced embryos. **b:** *Cyc* gene expression log2 fold changes and significance levels in sexually produced embryos. **c:** *Clk* gene expression log2 fold changes and significance levels in asexually produced embryos. **d**: *Cyc* gene expression log2 fold changes and significance levels in asexually produced embryos.


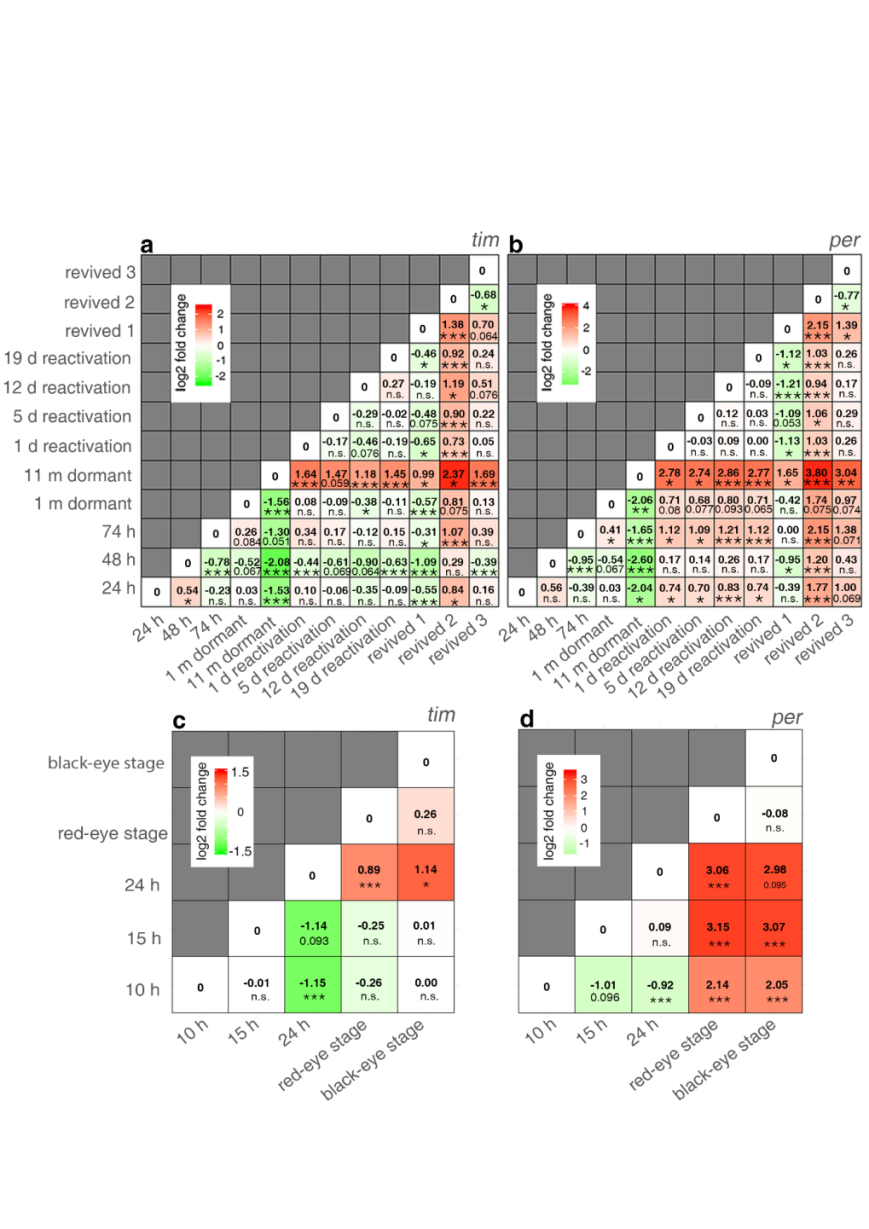


**Fig. S2:** Heatmaps (generated with the packages “ggplot2” and “reshape2” in R Version 1.1.383 [1,2,3], displaying the log2 fold changes of gene expression between all tested stages and the statistical results obtained from REST analysis) of *tim* and *per* mRNA expression patterns across developmental stages in sexually and asexually produced *D. magna* embryos. Displayed are the log2 fold expression change (top value) and significance level (bottom indicated significance level * *P*<0.05; ** *P*<0.01; *** *P*<0.001; n.s.= not significant when *P >0.1*) in the developmental stage depicted on the x-axis with respect to the stage on the y-axis. **a:** *Tim* gene expression log2 fold changes and significance levels in sexually produced embryos. **b:** *Per* gene expression log2 fold changes and significance levels in sexually produced embryos. **c:** *Tim* gene expression log2 fold changes and significance levels in asexually produced embryos. **d**: *Per* gene expression log2 fold changes and significance levels in asexually produced embryos.


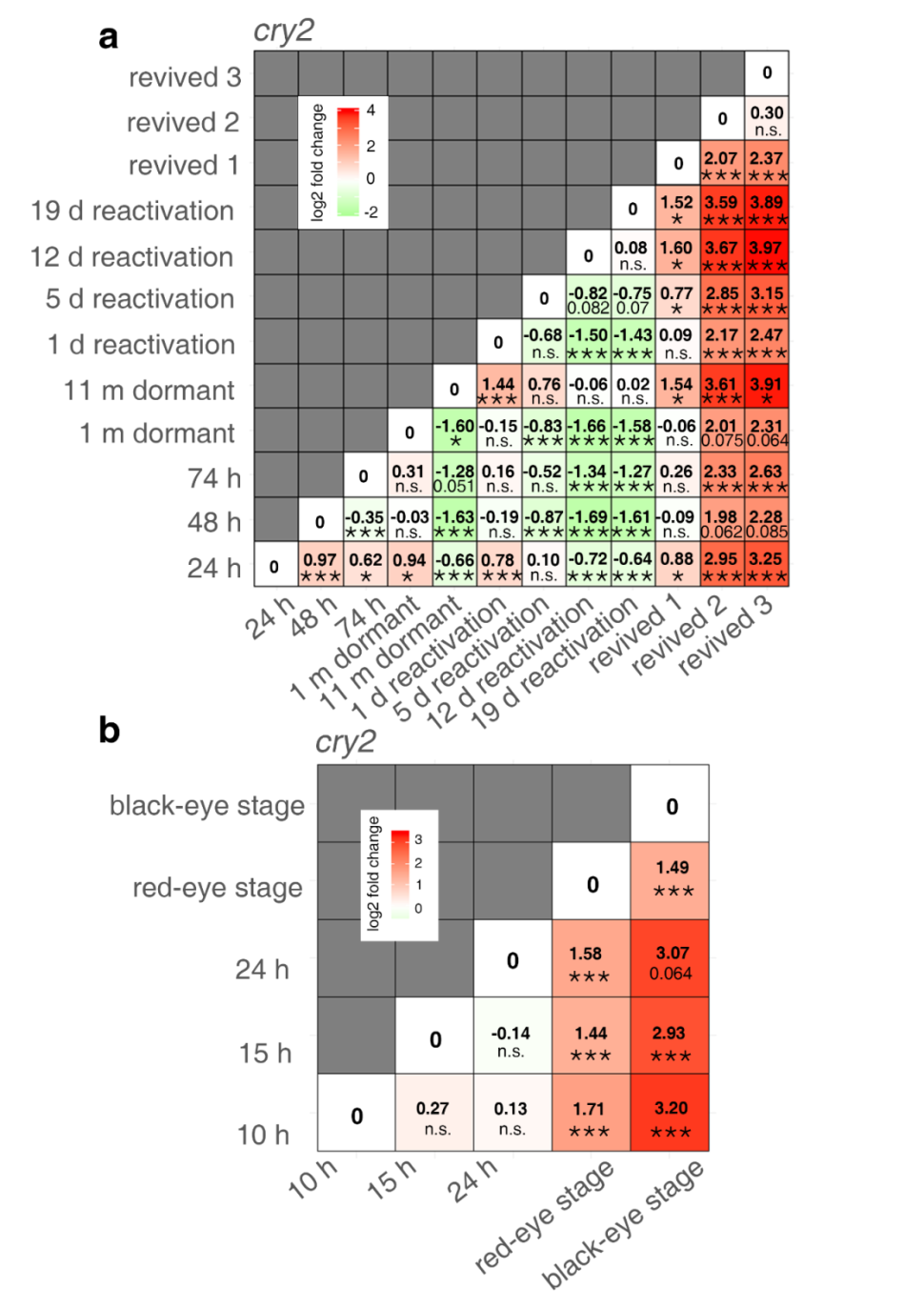


**Fig. S3:** Heatmaps (generated with the packages “ggplot2” and “reshape2” in R Version 1.1.383 [1,2,3], displaying the log2 fold changes of gene expression between all tested stages and the statistical results obtained from REST analysis) *cry2* mRNA expression patterns across developmental stages in sexually and asexually produced *D. magna* embryos. Displayed are the log2 fold expression change (top value) and significance level (bottom indicated significance level * *P*<0.05; ** *P*<0.01; *** *P*<0.001; n.s.= not significant when *P >0.1*) in the developmental stage depicted on the x-axis with respect to the stage on the y-axis. **a:** *Cry2* gene expression log2 fold changes and significance levels in sexually produced embryos. **b:** *Cry2* gene expression log2 fold changes and significance levels in asexually produced embryos.


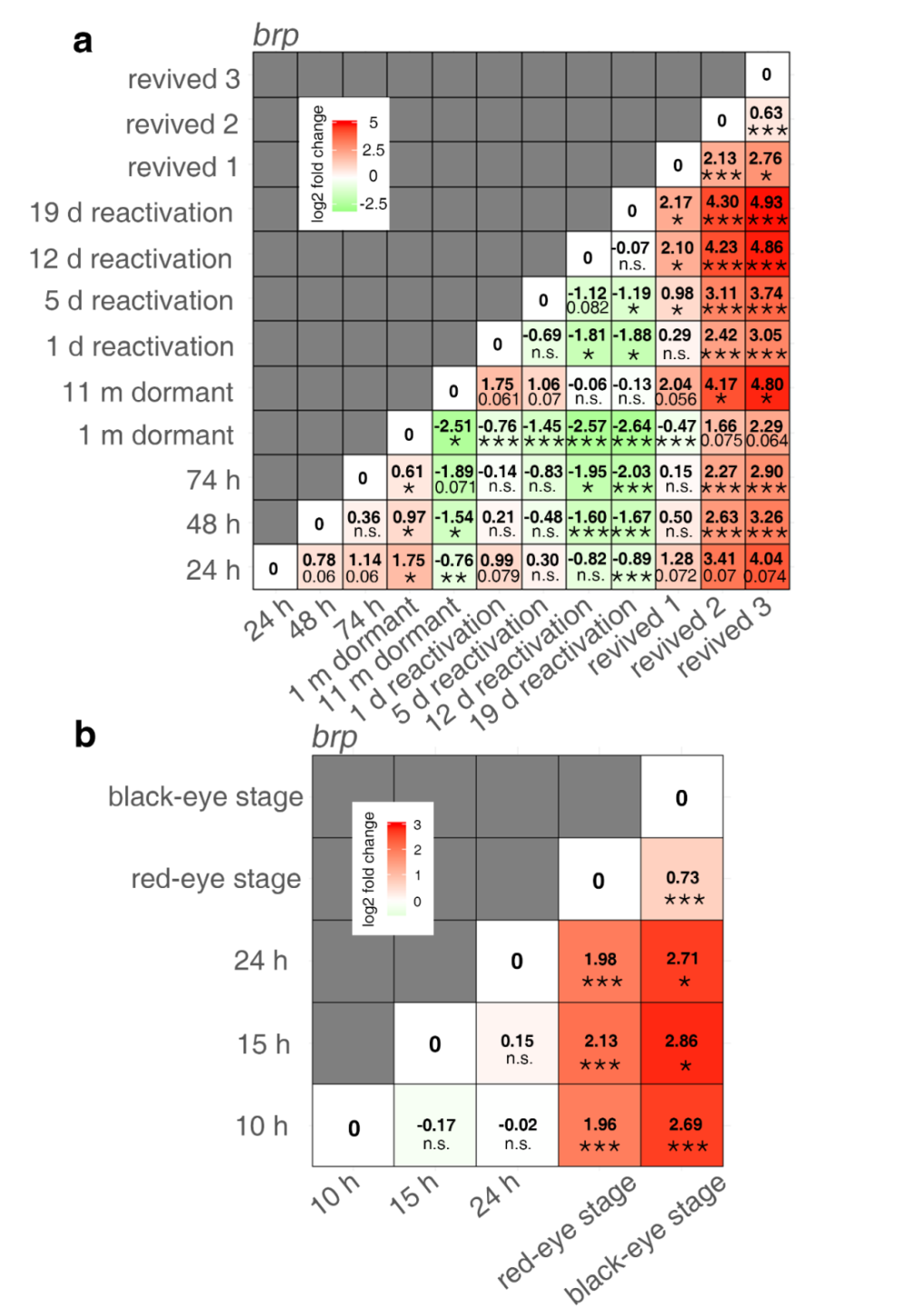


**Fig. S4:** Heatmaps (generated with the packages “ggplot2” and “reshape2” in R Version 1.1.383 [1,2,3], displaying the log2 fold changes of gene expression between all tested stages and the statistical results obtained from REST analysis) *brp* mRNA expression patterns across developmental stages in sexually and asexually produced *D. magna* embryos. Displayed are the log2 fold expression change (top value) and significance level (bottom indicated significance level * *P*<0.05; ** *P*<0.01; *** *P*<0.001; n.s.= not significant when *P >0.1*) in the developmental stage depicted on the x-axis with respect to the stage on the y-axis. **a:** *Brp* gene expression log2 fold changes and significance levels in sexually produced embryos. **b:** *Brp* gene expression log2 fold changes and significance levels in asexually produced embryos.

**Supplementary references:**

[1] R Core Team (2020). R: A language and environment for statistical computing. R Foundation for Statistical Computing, Vienna, Austria. URL

[2] H. Wickham. ggplot2: Elegant Graphics for Data Analysis. Springer-Verlag New York, 2016.

[3] Hadley Wickham (2007). Reshaping Data with the reshape Package. Journal of Statistical Software, 21(12), 1-20. URL <http://www.jstatsoft.org/v21/i12/>.

**Table S1:** Fold gene expression change and statistical results of sexually produced *Daphnia* embryos.

| **gene** | **Control stage** | **vs.** | **Treatement stage** | **fold expression** | **standard error** | **95% confidence interval** | **pValue** | **results** |
| --- | --- | --- | --- | --- | --- | --- | --- | --- |
| *clock* | 24 h | vs. | 48 h | 1.531 | 1.324 - 1.898 | 1.142 - 2.018 | 0.048 | UP |
| *clock* | 24 h | vs. | 74 h | 0.824 | 0.672 - 0.981 | 0.598 - 1.166 | 0.161 |  |
| *clock* | 24 h | vs. | 1 m dormant | 0.432 | 0.061 - 1.318 | 0.050 - 1.503 | 0.863 |  |
| *clock* | 24 h | vs. | 11 m dormant | 0.423 | 0.360 - 0.508 | 0.324 - 0.558 | 0 | DOWN |
| *clock* | 24 h | vs. | 1 d reactivation | 1.216 | 1.017 - 1.485 | 0.985 - 1.512 | 0.096 |  |
| *clock* | 24 h | vs. | 5 d reactivation | 0.902 | 0.742 - 1.088 | 0.648 - 1.282 | 0.467 |  |
| *clock* | 24 h | vs. | 12 d reactivation | 0.686 | 0.575 - 0.839 | 0.555 - 0.853 | 0.043 | DOWN |
| *clock* | 24 h | vs. | 19 d reactivation | 0.906 | 0.768 - 1.093 | 0.678 - 1.221 | 0.464 |  |
| *clock* | 24 h | vs. | revived 1 | 1.414 | 1.163 - 1.657 | 1.062 - 1.936 | 0.061 |  |
| *clock* | 24 h | vs. | revived 2 | 3.399 | 2.543 - 4.380 | 2.394 - 5.453 | 0.072 |  |
| *clock* | 24 h | vs. | revived 3 | 1.942 | 1.349 - 2.686 | 1.101 - 3.034 | 0.065 |  |
| *clock* | 48 h | vs. | 74 h | 0.538 | 0.446 - 0.606 | 0.427 - 0.718 | 0.034 | DOWN |
| *clock* | 48 h | vs. | 1 m dormant | 0.282 | 0.038 - 0.812 | 0.035 - 0.925 | 0.084 |  |
| *clock* | 48 h | vs. | 11 m dormant | 0.277 | 0.241 - 0.313 | 0.232 - 0.343 | 0 | DOWN |
| *clock* | 48 h | vs. | 1 d reactivation | 0.794 | 0.729 - 0.914 | 0.716 - 0.930 | 0.046 | DOWN |
| *clock* | 48 h | vs. | 5 d reactivation | 0.589 | 0.484 - 0.671 | 0.458 - 0.788 | 0.071 |  |
| *clock* | 48 h | vs. | 12 d reactivation | 0.448 | 0.411 - 0.516 | 0.404 - 0.525 | 0.055 |  |
| *clock* | 48 h | vs. | 19 d reactivation | 0.592 | 0.506 - 0.674 | 0.482 - 0.751 | 0 | DOWN |
| *clock* | 48 h | vs. | revived 1 | 0.923 | 0.788 - 1.021 | 0.766 - 1.191 | 0.494 |  |
| *clock* | 48 h | vs. | revived 2 | 2.22 | 1.769 - 2.830 | 1.749 - 3.359 | 0.065 |  |
| *clock* | 48 h | vs. | revived 3 | 1.268 | 0.834 - 1.656 | 0.777 - 1.866 | 0.342 |  |
| *clock* | 74 h | vs. | 1 m dormant | 0.525 | 0.075 - 1.569 | 0.062 - 1.748 | 0.95 |  |
| *clock* | 74 h | vs. | 11 m dormant | 0.514 | 0.441 - 0.599 | 0.396 - 0.648 | 0 | DOWN |
| *clock* | 74 h | vs. | 1 d reactivation | 1.475 | 1.241 - 1.725 | 1.202 - 1.757 | 0.037 | UP |
| *clock* | 74 h | vs. | 5 d reactivation | 1.095 | 0.913 - 1.331 | 0.791 - 1.505 | 0.533 |  |
| *clock* | 74 h | vs. | 12 d reactivation | 0.832 | 0.701 - 0.975 | 0.678 - 0.991 | 0.034 | DOWN |
| *clock* | 74 h | vs. | 19 d reactivation | 1.1 | 0.946 - 1.297 | 0.828 - 1.418 | 0.421 |  |
| *clock* | 74 h | vs. | revived 1 | 1.716 | 1.432 - 2.013 | 1.296 - 2.268 | 0.074 |  |
| *clock* | 74 h | vs. | revived 2 | 4.124 | 3.135 - 5.479 | 2.922 - 6.425 | 0.078 |  |
| *clock* | 74 h | vs. | revived 3 | 2.356 | 1.658 - 3.188 | 1.354 - 3.524 | 0.093 |  |
| *clock* | 1 m dormant | vs. | 11 m dormant | 0.979 | 0.342 - 7.127 | 0.311 - 7.822 | 0.914 |  |
| *clock* | 1 m dormant | vs. | 1 d reactivation | 2.811 | 0.975 - 20.825 | 0.944 - 21.207 | 0.368 |  |
| *clock* | 1 m dormant | vs. | 5 d reactivation | 2.086 | 0.693 - 14.690 | 0.616 - 17.971 | 0.946 |  |
| *clock* | 1 m dormant | vs. | 12 d reactivation | 1.586 | 0.551 - 11.765 | 0.532 - 11.964 | 0.986 |  |
| *clock* | 1 m dormant | vs. | 19 d reactivation | 2.096 | 0.725 - 15.198 | 0.649 - 17.123 | 0.981 |  |
| *clock* | 1 m dormant | vs. | revived 1 | 3.269 | 1.101 - 22.957 | 1.018 - 27.146 | 0.092 |  |
| *clock* | 1 m dormant | vs. | revived 2 | 7.86 | 2.408 - 50.104 | 2.295 - 73.677 | 0.096 |  |
| *clock* | 1 m dormant | vs. | revived 3 | 4.49 | 1.334 - 34.075 | 1.046 - 42.542 | 0.077 |  |
| *clock* | 11 m dormant | vs. | 1 d reactivation | 2.871 | 2.655 - 3.170 | 2.571 - 3.228 | 0 | UP |
| *clock* | 11 m dormant | vs. | 5 d reactivation | 2.131 | 1.811 - 2.487 | 1.658 - 2.777 | 0 | UP |
| *clock* | 11 m dormant | vs. | 12 d reactivation | 1.62 | 1.500 - 1.791 | 1.450 - 1.821 | 0 | UP |
| *clock* | 11 m dormant | vs. | 19 d reactivation | 2.141 | 1.894 - 2.396 | 1.745 - 2.607 | 0.102 |  |
| *clock* | 11 m dormant | vs. | revived 1 | 3.339 | 2.951 - 3.757 | 2.772 - 4.184 | 0.069 |  |
| *clock* | 11 m dormant | vs. | revived 2 | 8.028 | 6.448 - 10.614 | 6.251 - 11.851 | 0.063 |  |
| *clock* | 11 m dormant | vs. | revived 3 | 4.586 | 3.091 - 5.918 | 2.812 - 6.488 | 0.032 | UP |
| *clock* | 1 d reactivation | vs. | 5 d reactivation | 0.742 | 0.622 - 0.883 | 0.611 - 0.912 | 0.067 |  |
| *clock* | 1 d reactivation | vs. | 12 d reactivation | 0.564 | 0.548 - 0.582 | 0.539 - 0.590 | 0 | DOWN |
| *clock* | 1 d reactivation | vs. | 19 d reactivation | 0.746 | 0.655 - 0.828 | 0.643 - 0.855 | 0 | DOWN |
| *clock* | 1 d reactivation | vs. | revived 1 | 1.163 | 1.040 - 1.331 | 1.021 - 1.375 | 0.084 |  |
| *clock* | 1 d reactivation | vs. | revived 2 | 2.796 | 2.357 - 3.770 | 2.334 - 3.894 | 0.065 |  |
| *clock* | 1 d reactivation | vs. | revived 3 | 1.597 | 1.056 - 2.064 | 1.036 - 2.132 | 0.061 |  |
| *clock* | 5 d reactivation | vs. | 12 d reactivation | 0.76 | 0.640 - 0.908 | 0.618 - 0.923 | 0 | DOWN |
| *clock* | 5 d reactivation | vs. | 19 d reactivation | 1.005 | 0.859 - 1.196 | 0.755 - 1.322 | 0.982 |  |
| *clock* | 5 d reactivation | vs. | revived 1 | 1.567 | 1.300 - 1.825 | 1.182 - 2.099 | 0.087 |  |
| *clock* | 5 d reactivation | vs. | revived 2 | 3.768 | 2.845 - 4.935 | 2.666 - 5.947 | 0.086 |  |
| *clock* | 5 d reactivation | vs. | revived 3 | 2.152 | 1.504 - 2.941 | 1.230 - 3.284 | 0.073 |  |
| *clock* | 12 d reactivation | vs. | 19 d reactivation | 1.321 | 1.159 - 1.465 | 1.140 - 1.516 | 0.053 |  |
| *clock* | 12 d reactivation | vs. | revived 1 | 2.061 | 1.842 - 2.356 | 1.811 - 2.438 | 0.074 |  |
| *clock* | 12 d reactivation | vs. | revived 2 | 4.955 | 4.172 - 6.673 | 4.136 - 6.904 | 0.063 |  |
| *clock* | 12 d reactivation | vs. | revived 3 | 2.83 | 1.868 - 3.653 | 1.837 - 3.780 | 0.039 | UP |
| *clock* | 19 d reactivation | vs. | revived 1 | 1.56 | 1.351 - 1.763 | 1.261 - 2.003 | 0.068 |  |
| *clock* | 19 d reactivation | vs. | revived 2 | 3.75 | 2.952 - 4.907 | 2.844 - 5.672 | 0.055 |  |
| *clock* | 19 d reactivation | vs. | revived 3 | 2.142 | 1.452 - 2.811 | 1.286 - 3.120 | 0.041 | UP |
| *clock* | revived 1 | vs. | revived 2 | 2.404 | 1.880 - 3.240 | 1.769 - 3.615 | 0.045 | UP |
| *clock* | revived 1 | vs. | revived 3 | 1.373 | 0.958 - 1.836 | 0.814 - 1.979 | 0.223 |  |
| *clock* | revived 2 | vs. | revived 3 | 0.571 | 0.439 - 0.842 | 0.308 - 0.877 | 0.077 |  |
| *cycle* | 24 h | vs. | 48 h | 3.552 | 2.938 - 3.991 | 2.842 - 4.839 | 0.026 | UP |
| *cycle* | 24 h | vs. | 74 h | 3.362 | 2.784 - 4.149 | 2.479 - 4.523 | 0.02 | UP |
| *cycle* | 24 h | vs. | 1 m dormant | 2.571 | 2.087 - 2.956 | 2.001 - 3.654 | 0.021 | UP |
| *cycle* | 24 h | vs. | 11 m dormant | 0.727 | 0.581 - 0.877 | 0.523 - 1.036 | 0.185 |  |
| *cycle* | 24 h | vs. | 1 d reactivation | 3.483 | 3.056 - 4.216 | 3.005 - 4.249 | 0.021 | UP |
| *cycle* | 24 h | vs. | 5 d reactivation | 3.347 | 2.723 - 3.786 | 2.657 - 4.666 | 0.061 |  |
| *cycle* | 24 h | vs. | 12 d reactivation | 2.703 | 2.271 - 3.249 | 2.065 - 3.609 | 0.062 |  |
| *cycle* | 24 h | vs. | 19 d reactivation | 2.347 | 1.993 - 2.750 | 1.848 - 3.132 | 0.029 | UP |
| *cycle* | 24 h | vs. | revived 1 | 2.213 | 1.917 - 2.609 | 1.791 - 2.890 | 0.095 |  |
| *cycle* | 24 h | vs. | revived 2 | 9.425 | 6.976 - 12.821 | 6.414 - 16.008 | 0.062 |  |
| *cycle* | 24 h | vs. | revived 3 | 10.246 | 8.142 - 13.014 | 7.085 - 14.288 | 0.024 | UP |
| *cycle* | 48 h | vs. | 74 h | 0.946 | 0.849 - 1.131 | 0.705 - 1.157 | 0.621 |  |
| *cycle* | 48 h | vs. | 1 m dormant | 0.724 | 0.600 - 0.900 | 0.550 - 0.968 | 0.067 |  |
| *cycle* | 48 h | vs. | 11 m dormant | 0.205 | 0.177 - 0.257 | 0.148 - 0.272 | 0 | DOWN |
| *cycle* | 48 h | vs. | 1 d reactivation | 0.98 | 0.840 - 1.074 | 0.826 - 1.081 | 0.969 |  |
| *cycle* | 48 h | vs. | 5 d reactivation | 0.942 | 0.783 - 1.149 | 0.731 - 1.236 | 0.392 |  |
| *cycle* | 48 h | vs. | 12 d reactivation | 0.761 | 0.680 - 0.900 | 0.581 - 0.929 | 0.033 | DOWN |
| *cycle* | 48 h | vs. | 19 d reactivation | 0.661 | 0.580 - 0.779 | 0.512 - 0.813 | 0 | DOWN |
| *cycle* | 48 h | vs. | revived 1 | 0.623 | 0.544 - 0.720 | 0.493 - 0.747 | 0 | DOWN |
| *cycle* | 48 h | vs. | revived 2 | 2.653 | 2.056 - 3.943 | 1.788 - 4.242 | 0.034 | UP |
| *cycle* | 48 h | vs. | revived 3 | 2.884 | 2.426 - 3.571 | 2.015 - 3.663 | 0.069 |  |
| *cycle* | 74 h | vs. | 1 m dormant | 0.765 | 0.628 - 0.867 | 0.609 - 1.078 | 0.178 |  |
| *cycle* | 74 h | vs. | 11 m dormant | 0.216 | 0.172 - 0.259 | 0.158 - 0.306 | 0 | DOWN |
| *cycle* | 74 h | vs. | 1 d reactivation | 1.036 | 0.930 - 1.246 | 0.914 - 1.256 | 0.945 |  |
| *cycle* | 74 h | vs. | 5 d reactivation | 0.995 | 0.819 - 1.111 | 0.808 - 1.377 | 0.923 |  |
| *cycle* | 74 h | vs. | 12 d reactivation | 0.804 | 0.672 - 0.958 | 0.623 - 1.067 | 0.264 |  |
| *cycle* | 74 h | vs. | 19 d reactivation | 0.698 | 0.590 - 0.813 | 0.558 - 0.926 | 0 | DOWN |
| *cycle* | 74 h | vs. | revived 1 | 0.658 | 0.567 - 0.771 | 0.541 - 0.854 | 0.032 | DOWN |
| *cycle* | 74 h | vs. | revived 2 | 2.803 | 2.064 - 3.789 | 1.935 - 4.725 | 0.057 |  |
| *cycle* | 74 h | vs. | revived 3 | 3.048 | 2.390 - 3.838 | 2.138 - 4.224 | 0.029 | UP |
| *cycle* | 1 m dormant | vs. | 11 m dormant | 0.283 | 0.238 - 0.361 | 0.196 - 0.386 | 0 | DOWN |
| *cycle* | 1 m dormant | vs. | 1 d reactivation | 1.355 | 1.111 - 1.529 | 1.092 - 1.541 | 0.059 |  |
| *cycle* | 1 m dormant | vs. | 5 d reactivation | 1.302 | 1.052 - 1.606 | 0.966 - 1.756 | 0.28 |  |
| *cycle* | 1 m dormant | vs. | 12 d reactivation | 1.051 | 0.907 - 1.267 | 0.768 - 1.319 | 0.854 |  |
| *cycle* | 1 m dormant | vs. | 19 d reactivation | 0.913 | 0.769 - 1.096 | 0.676 - 1.155 | 0.512 |  |
| *cycle* | 1 m dormant | vs. | revived 1 | 0.861 | 0.719 - 1.013 | 0.652 - 1.060 | 0.202 |  |
| *cycle* | 1 m dormant | vs. | revived 2 | 3.666 | 2.764 - 5.452 | 2.364 - 6.025 | 0.086 |  |
| *cycle* | 1 m dormant | vs. | revived 3 | 3.985 | 3.414 - 5.024 | 2.701 - 5.202 | 0.064 |  |
| *cycle* | 11 m dormant | vs. | 1 d reactivation | 4.789 | 3.954 - 5.908 | 3.889 - 5.954 | 0 | UP |
| *cycle* | 11 m dormant | vs. | 5 d reactivation | 4.601 | 3.633 - 5.395 | 3.438 - 6.563 | 0.028 | UP |
| *cycle* | 11 m dormant | vs. | 12 d reactivation | 3.716 | 3.161 - 4.578 | 2.733 - 5.057 | 0 | UP |
| *cycle* | 11 m dormant | vs. | 19 d reactivation | 3.227 | 2.694 - 3.872 | 2.408 - 4.390 | 0 | UP |
| *cycle* | 11 m dormant | vs. | revived 1 | 3.043 | 2.558 - 3.656 | 2.320 - 4.050 | 0.041 | UP |
| *cycle* | 11 m dormant | vs. | revived 2 | 12.959 | 9.549 - 17.920 | 8.415 - 22.518 | 0.035 | UP |
| *cycle* | 11 m dormant | vs. | revived 3 | 14.087 | 11.362 - 18.336 | 9.380 - 20.023 | 0.032 | UP |
| *cycle* | 1 d reactivation | vs. | 5 d reactivation | 0.961 | 0.869 - 1.150 | 0.868 - 1.169 | 0.724 |  |
| *cycle* | 1 d reactivation | vs. | 12 d reactivation | 0.776 | 0.668 - 0.864 | 0.663 - 0.878 | 0.051 |  |
| *cycle* | 1 d reactivation | vs. | 19 d reactivation | 0.674 | 0.598 - 0.756 | 0.593 - 0.769 | 0 | DOWN |
| *cycle* | 1 d reactivation | vs. | revived 1 | 0.636 | 0.580 - 0.694 | 0.575 - 0.706 | 0 | DOWN |
| *cycle* | 1 d reactivation | vs. | revived 2 | 2.706 | 2.076 - 3.945 | 2.060 - 4.011 | 0.05 | UP |
| *cycle* | 1 d reactivation | vs. | revived 3 | 2.942 | 2.293 - 3.406 | 2.275 - 3.464 | 0.028 | UP |
| *cycle* | 5 d reactivation | vs. | 12 d reactivation | 0.808 | 0.710 - 0.972 | 0.601 - 0.993 | 0.065 |  |
| *cycle* | 5 d reactivation | vs. | 19 d reactivation | 0.701 | 0.602 - 0.841 | 0.530 - 0.869 | 0.033 | DOWN |
| *cycle* | 5 d reactivation | vs. | revived 1 | 0.661 | 0.563 - 0.777 | 0.510 - 0.798 | 0.075 |  |
| *cycle* | 5 d reactivation | vs. | revived 2 | 2.816 | 2.157 - 4.231 | 1.851 - 4.537 | 0.04 | UP |
| *cycle* | 5 d reactivation | vs. | revived 3 | 3.062 | 2.618 - 3.854 | 2.109 - 3.917 | 0 | UP |
| *cycle* | 12 d reactivation | vs. | 19 d reactivation | 0.868 | 0.752 - 0.990 | 0.699 - 1.112 | 0.196 |  |
| *cycle* | 12 d reactivation | vs. | revived 1 | 0.819 | 0.723 - 0.926 | 0.678 - 1.026 | 0.124 |  |
| *cycle* | 12 d reactivation | vs. | revived 2 | 3.487 | 2.631 - 4.835 | 2.427 - 5.739 | 0.054 |  |
| *cycle* | 12 d reactivation | vs. | revived 3 | 3.791 | 3.009 - 4.686 | 2.681 - 5.072 | 0.035 | UP |
| *cycle* | 19 d reactivation | vs. | revived 1 | 0.943 | 0.841 - 1.054 | 0.776 - 1.147 | 0.536 |  |
| *cycle* | 19 d reactivation | vs. | revived 2 | 4.016 | 3.107 - 5.716 | 2.801 - 6.491 | 0.032 | UP |
| *cycle* | 19 d reactivation | vs. | revived 3 | 4.365 | 3.525 - 5.328 | 3.094 - 5.668 | 0 | UP |
| *cycle* | revived 1 | vs. | revived 2 | 4.258 | 3.322 - 6.108 | 3.033 - 6.731 | 0.022 | UP |
| *cycle* | revived 1 | vs. | revived 3 | 4.629 | 3.712 - 5.537 | 3.350 - 5.846 | 0 | UP |
| *cycle* | revived 2 | vs. | revived 3 | 1.087 | 0.829 - 1.523 | 0.625 - 1.633 | 0.81 |  |
| *tim* | 24 h | vs. | 48 h | 1.457 | 1.186 - 1.751 | 1.051 - 1.873 | 0.035 | UP |
| *tim* | 24 h | vs. | 74 h | 0.85 | 0.667 - 1.065 | 0.604 - 1.191 | 0.409 |  |
| *tim* | 24 h | vs. | 1 m dormant | 1.018 | 0.838 - 1.218 | 0.744 - 1.264 | 0.918 |  |
| *tim* | 24 h | vs. | 11 m dormant | 0.346 | 0.280 - 0.424 | 0.245 - 0.461 | 0 | DOWN |
| *tim* | 24 h | vs. | 1 d reactivation | 1.075 | 0.845 - 1.316 | 0.777 - 1.452 | 0.523 |  |
| *tim* | 24 h | vs. | 5 d reactivation | 0.958 | 0.768 - 1.197 | 0.674 - 1.320 | 0.494 |  |
| *tim* | 24 h | vs. | 12 d reactivation | 0.783 | 0.639 - 0.965 | 0.552 - 1.046 | 0.16 |  |
| *tim* | 24 h | vs. | 19 d reactivation | 0.942 | 0.762 - 1.142 | 0.677 - 1.233 | 0.642 |  |
| *tim* | 24 h | vs. | revived 1 | 0.685 | 0.553 - 0.804 | 0.521 - 0.822 | 0 | DOWN |
| *tim* | 24 h | vs. | revived 2 | 1.787 | 1.304 - 2.677 | 1.108 - 3.065 | 0.011 | UP |
| *tim* | 24 h | vs. | revived 3 | 1.114 | 0.917 - 1.374 | 0.782 - 1.482 | 0.427 |  |
| *tim* | 48 h | vs. | 74 h | 0.583 | 0.490 - 0.689 | 0.472 - 0.777 | 0 | DOWN |
| *tim* | 48 h | vs. | 1 m dormant | 0.698 | 0.634 - 0.792 | 0.577 - 0.831 | 0.067 |  |
| *tim* | 48 h | vs. | 11 m dormant | 0.237 | 0.208 - 0.270 | 0.191 - 0.301 | 0 | DOWN |
| *tim* | 48 h | vs. | 1 d reactivation | 0.737 | 0.631 - 0.840 | 0.606 - 0.947 | 0 | DOWN |
| *tim* | 48 h | vs. | 5 d reactivation | 0.657 | 0.564 - 0.763 | 0.526 - 0.861 | 0.069 |  |
| *tim* | 48 h | vs. | 12 d reactivation | 0.537 | 0.469 - 0.615 | 0.428 - 0.682 | 0.064 |  |
| *tim* | 48 h | vs. | 19 d reactivation | 0.647 | 0.572 - 0.728 | 0.528 - 0.804 | 0 | DOWN |
| *tim* | 48 h | vs. | revived 1 | 0.47 | 0.431 - 0.527 | 0.407 - 0.540 | 0 | DOWN |
| *tim* | 48 h | vs. | revived 2 | 1.226 | 0.944 - 1.773 | 0.859 - 1.999 | 0.384 |  |
| *tim* | 48 h | vs. | revived 3 | 0.764 | 0.666 - 0.875 | 0.604 - 0.967 | 0 | DOWN |
| *tim* | 74 h | vs. | 1 m dormant | 1.197 | 1.028 - 1.397 | 0.913 - 1.421 | 0.084 |  |
| *tim* | 74 h | vs. | 11 m dormant | 0.407 | 0.344 - 0.496 | 0.301 - 0.522 | 0.051 |  |
| *tim* | 74 h | vs. | 1 d reactivation | 1.264 | 1.036 - 1.539 | 0.953 - 1.644 | 0.201 |  |
| *tim* | 74 h | vs. | 5 d reactivation | 1.127 | 0.936 - 1.399 | 0.827 - 1.494 | 0.481 |  |
| *tim* | 74 h | vs. | 12 d reactivation | 0.921 | 0.784 - 1.128 | 0.677 - 1.185 | 0.554 |  |
| *tim* | 74 h | vs. | 19 d reactivation | 1.109 | 0.935 - 1.334 | 0.830 - 1.396 | 0.399 |  |
| *tim* | 74 h | vs. | revived 1 | 0.806 | 0.678 - 0.913 | 0.639 - 0.920 | 0.041 | DOWN |
| *tim* | 74 h | vs. | revived 2 | 2.102 | 1.583 - 3.197 | 1.360 - 3.470 | 0 | UP |
| *tim* | 74 h | vs. | revived 3 | 1.311 | 1.124 - 1.605 | 0.959 - 1.678 | 0.164 |  |
| *tim* | 1 m dormant | vs. | 11 m dormant | 0.34 | 0.295 - 0.377 | 0.281 - 0.425 | 0 | DOWN |
| *tim* | 1 m dormant | vs. | 1 d reactivation | 1.056 | 0.923 - 1.186 | 0.905 - 1.339 | 0.665 |  |
| *tim* | 1 m dormant | vs. | 5 d reactivation | 0.941 | 0.809 - 1.078 | 0.777 - 1.217 | 0.513 |  |
| *tim* | 1 m dormant | vs. | 12 d reactivation | 0.769 | 0.664 - 0.858 | 0.629 - 0.965 | 0.032 | DOWN |
| *tim* | 1 m dormant | vs. | 19 d reactivation | 0.926 | 0.813 - 1.015 | 0.777 - 1.137 | 0.396 |  |
| *tim* | 1 m dormant | vs. | revived 1 | 0.673 | 0.625 - 0.749 | 0.602 - 0.767 | 0 | DOWN |
| *tim* | 1 m dormant | vs. | revived 2 | 1.756 | 1.335 - 2.503 | 1.262 - 2.827 | 0.075 |  |
| *tim* | 1 m dormant | vs. | revived 3 | 1.095 | 0.943 - 1.221 | 0.887 - 1.367 | 0.374 |  |
| *tim* | 11 m dormant | vs. | 1 d reactivation | 3.11 | 2.610 - 3.605 | 2.464 - 4.060 | 0 | UP |
| *tim* | 11 m dormant | vs. | 5 d reactivation | 2.772 | 2.332 - 3.278 | 2.136 - 3.691 | 0.059 |  |
| *tim* | 11 m dormant | vs. | 12 d reactivation | 2.265 | 1.956 - 2.645 | 1.749 - 2.926 | 0 | UP |
| *tim* | 11 m dormant | vs. | 19 d reactivation | 2.727 | 2.364 - 3.131 | 2.146 - 3.447 | 0 | UP |
| *tim* | 11 m dormant | vs. | revived 1 | 1.982 | 1.752 - 2.258 | 1.652 - 2.313 | 0.041 | UP |
| *tim* | 11 m dormant | vs. | revived 2 | 5.171 | 3.950 - 7.529 | 3.514 - 8.570 | 0.018 | UP |
| *tim* | 11 m dormant | vs. | revived 3 | 3.224 | 2.805 - 3.765 | 2.478 - 4.144 | 0 | UP |
| *tim* | 1 d reactivation | vs. | 5 d reactivation | 0.891 | 0.754 - 1.085 | 0.678 - 1.163 | 0.334 |  |
| *tim* | 1 d reactivation | vs. | 12 d reactivation | 0.728 | 0.632 - 0.875 | 0.555 - 0.922 | 0.076 |  |
| *tim* | 1 d reactivation | vs. | 19 d reactivation | 0.877 | 0.764 - 1.035 | 0.681 - 1.086 | 0.358 |  |
| *tim* | 1 d reactivation | vs. | revived 1 | 0.637 | 0.556 - 0.710 | 0.524 - 0.716 | 0.035 | DOWN |
| *tim* | 1 d reactivation | vs. | revived 2 | 1.663 | 1.277 - 2.509 | 1.115 - 2.700 | 0 | UP |
| *tim* | 1 d reactivation | vs. | revived 3 | 1.037 | 0.906 - 1.245 | 0.787 - 1.305 | 0.819 |  |
| *tim* | 5 d reactivation | vs. | 12 d reactivation | 0.817 | 0.695 - 0.977 | 0.611 - 1.064 | 0.185 |  |
| *tim* | 5 d reactivation | vs. | 19 d reactivation | 0.984 | 0.841 - 1.156 | 0.749 - 1.253 | 1 |  |
| *tim* | 5 d reactivation | vs. | revived 1 | 0.715 | 0.612 - 0.817 | 0.577 - 0.836 | 0.075 |  |
| *tim* | 5 d reactivation | vs. | revived 2 | 1.866 | 1.404 - 2.759 | 1.227 - 3.116 | 0 | UP |
| *tim* | 5 d reactivation | vs. | revived 3 | 1.163 | 0.997 - 1.390 | 0.865 - 1.507 | 0.215 |  |
| *tim* | 12 d reactivation | vs. | 19 d reactivation | 1.204 | 1.039 - 1.377 | 0.945 - 1.534 | 0.185 |  |
| *tim* | 12 d reactivation | vs. | revived 1 | 0.875 | 0.772 - 1.008 | 0.728 - 1.033 | 0.204 |  |
| *tim* | 12 d reactivation | vs. | revived 2 | 2.283 | 1.736 - 3.298 | 1.548 - 3.813 | 0.025 | UP |
| *tim* | 12 d reactivation | vs. | revived 3 | 1.423 | 1.233 - 1.656 | 1.092 - 1.844 | 0.076 |  |
| *tim* | 19 d reactivation | vs. | revived 1 | 0.727 | 0.655 - 0.816 | 0.618 - 0.836 | 0.029 | DOWN |
| *tim* | 19 d reactivation | vs. | revived 2 | 1.896 | 1.467 - 2.764 | 1.314 - 3.104 | 0 | UP |
| *tim* | 19 d reactivation | vs. | revived 3 | 1.182 | 1.037 - 1.371 | 0.924 - 1.501 | 0.103 |  |
| *tim* | revived 1 | vs. | revived 2 | 2.609 | 1.991 - 3.795 | 1.942 - 4.029 | 0 | UP |
| *tim* | revived 1 | vs. | revived 3 | 1.626 | 1.399 - 1.835 | 1.365 - 1.948 | 0.064 |  |
| *tim* | revived 2 | vs. | revived 3 | 0.623 | 0.437 - 0.820 | 0.373 - 0.918 | 0.016 | DOWN |
| *period* | 24 h | vs. | 48 h | 1.479 | 1.187 - 1.819 | 1.030 - 2.158 | 0.113 |  |
| *period* | 24 h | vs. | 74 h | 0.765 | 0.634 - 0.924 | 0.584 - 0.993 | 0.195 |  |
| *period* | 24 h | vs. | 1 m dormant | 1.02 | 0.832 - 1.234 | 0.724 - 1.440 | 0.838 |  |
| *period* | 24 h | vs. | 11 m dormant | 0.244 | 0.202 - 0.292 | 0.180 - 0.330 | 0.02 | DOWN |
| *period* | 24 h | vs. | 1 d reactivation | 1.668 | 1.383 - 2.135 | 1.142 - 2.321 | 0.033 | UP |
| *period* | 24 h | vs. | 5 d reactivation | 1.629 | 1.279 - 2.088 | 1.061 - 2.480 | 0.036 | UP |
| *period* | 24 h | vs. | 12 d reactivation | 1.773 | 1.477 - 2.228 | 1.235 - 2.473 | 0 | UP |
| *period* | 24 h | vs. | 19 d reactivation | 1.665 | 1.276 - 2.072 | 1.173 - 2.539 | 0.042 | UP |
| *period* | 24 h | vs. | revived 1 | 0.764 | 0.630 - 0.907 | 0.566 - 1.036 | 0.168 |  |
| *period* | 24 h | vs. | revived 2 | 3.399 | 2.551 - 4.497 | 2.253 - 5.581 | 0 | UP |
| *period* | 24 h | vs. | revived 3 | 1.997 | 1.635 - 2.509 | 1.365 - 2.853 | 0.069 |  |
| *period* | 48 h | vs. | 74 h | 0.517 | 0.429 - 0.613 | 0.396 - 0.659 | 0 | DOWN |
| *period* | 48 h | vs. | 1 m dormant | 0.69 | 0.566 - 0.836 | 0.490 - 0.959 | 0.067 |  |
| *period* | 48 h | vs. | 11 m dormant | 0.165 | 0.137 - 0.195 | 0.122 - 0.219 | 0 | DOWN |
| *period* | 48 h | vs. | 1 d reactivation | 1.128 | 0.947 - 1.430 | 0.776 - 1.540 | 0.521 |  |
| *period* | 48 h | vs. | 5 d reactivation | 1.102 | 0.876 - 1.423 | 0.721 - 1.656 | 0.638 |  |
| *period* | 48 h | vs. | 12 d reactivation | 1.199 | 1.012 - 1.492 | 0.840 - 1.641 | 0.148 |  |
| *period* | 48 h | vs. | 19 d reactivation | 1.126 | 0.868 - 1.414 | 0.794 - 1.695 | 0.531 |  |
| *period* | 48 h | vs. | revived 1 | 0.517 | 0.428 - 0.608 | 0.383 - 0.688 | 0.038 | DOWN |
| *period* | 48 h | vs. | revived 2 | 2.298 | 1.735 - 3.084 | 1.525 - 3.727 | 0 | UP |
| *period* | 48 h | vs. | revived 3 | 1.351 | 1.120 - 1.681 | 0.927 - 1.893 | 0.169 |  |
| *period* | 74 h | vs. | 1 m dormant | 1.333 | 1.149 - 1.552 | 1.069 - 1.685 | 0.016 | UP |
| *period* | 74 h | vs. | 11 m dormant | 0.318 | 0.285 - 0.354 | 0.268 - 0.383 | 0 | DOWN |
| *period* | 74 h | vs. | 1 d reactivation | 2.18 | 1.787 - 2.526 | 1.662 - 2.665 | 0.036 | UP |
| *period* | 74 h | vs. | 5 d reactivation | 2.129 | 1.660 - 2.679 | 1.544 - 2.908 | 0.034 | UP |
| *period* | 74 h | vs. | 12 d reactivation | 2.317 | 1.933 - 2.657 | 1.798 - 2.848 | 0 | UP |
| *period* | 74 h | vs. | 19 d reactivation | 2.176 | 1.837 - 2.742 | 1.767 - 2.977 | 0 | UP |
| *period* | 74 h | vs. | revived 1 | 0.999 | 0.897 - 1.110 | 0.847 - 1.205 | 0.943 |  |
| *period* | 74 h | vs. | revived 2 | 4.442 | 3.582 - 6.028 | 3.350 - 6.544 | 0 | UP |
| *period* | 74 h | vs. | revived 3 | 2.611 | 2.136 - 3.054 | 1.986 - 3.312 | 0.071 |  |
| *period* | 1 m dormant | vs. | 11 m dormant | 0.239 | 0.203 - 0.278 | 0.183 - 0.311 | 0.005 | DOWN |
| *period* | 1 m dormant | vs. | 1 d reactivation | 1.636 | 1.374 - 2.034 | 1.152 - 2.187 | 0.08 |  |
| *period* | 1 m dormant | vs. | 5 d reactivation | 1.598 | 1.271 - 2.032 | 1.071 - 2.355 | 0.077 |  |
| *period* | 1 m dormant | vs. | 12 d reactivation | 1.739 | 1.467 - 2.124 | 1.247 - 2.331 | 0.093 |  |
| *period* | 1 m dormant | vs. | 19 d reactivation | 1.633 | 1.284 - 2.029 | 1.193 - 2.411 | 0.065 |  |
| *period* | 1 m dormant | vs. | revived 1 | 0.749 | 0.634 - 0.865 | 0.575 - 0.977 | 0.105 |  |
| *period* | 1 m dormant | vs. | revived 2 | 3.333 | 2.570 - 4.460 | 2.291 - 5.300 | 0.075 |  |
| *period* | 1 m dormant | vs. | revived 3 | 1.959 | 1.624 - 2.392 | 1.377 - 2.689 | 0.074 |  |
| *period* | 11 m dormant | vs. | 1 d reactivation | 6.846 | 5.735 - 8.213 | 5.008 - 8.733 | 0.035 | UP |
| *period* | 11 m dormant | vs. | 5 d reactivation | 6.686 | 5.328 - 8.382 | 4.653 - 9.478 | 0.033 | UP |
| *period* | 11 m dormant | vs. | 12 d reactivation | 7.276 | 6.204 - 8.577 | 5.418 - 9.307 | 0 | UP |
| *period* | 11 m dormant | vs. | 19 d reactivation | 6.832 | 5.561 - 8.572 | 5.247 - 9.701 | 0 | UP |
| *period* | 11 m dormant | vs. | revived 1 | 3.136 | 2.747 - 3.555 | 2.529 - 3.926 | 0.023 | UP |
| *period* | 11 m dormant | vs. | revived 2 | 13.946 | 11.138 - 18.845 | 10.078 - 21.326 | 0 | UP |
| *period* | 11 m dormant | vs. | revived 3 | 8.197 | 6.853 - 9.793 | 5.985 - 10.793 | 0.007 | UP |
| *period* | 1 d reactivation | vs. | 5 d reactivation | 0.977 | 0.739 - 1.210 | 0.661 - 1.493 | 0.933 |  |
| *period* | 1 d reactivation | vs. | 12 d reactivation | 1.063 | 0.852 - 1.319 | 0.769 - 1.499 | 0.486 |  |
| *period* | 1 d reactivation | vs. | 19 d reactivation | 0.998 | 0.786 - 1.171 | 0.757 - 1.515 | 1 |  |
| *period* | 1 d reactivation | vs. | revived 1 | 0.458 | 0.383 - 0.544 | 0.363 - 0.628 | 0.028 | DOWN |
| *period* | 1 d reactivation | vs. | revived 2 | 2.037 | 1.531 - 2.575 | 1.434 - 3.330 | 0 | UP |
| *period* | 1 d reactivation | vs. | revived 3 | 1.197 | 0.944 - 1.484 | 0.850 - 1.729 | 0.313 |  |
| *period* | 5 d reactivation | vs. | 12 d reactivation | 1.088 | 0.883 - 1.425 | 0.731 - 1.614 | 0.628 |  |
| *period* | 5 d reactivation | vs. | 19 d reactivation | 1.022 | 0.756 - 1.272 | 0.691 - 1.634 | 0.794 |  |
| *period* | 5 d reactivation | vs. | revived 1 | 0.469 | 0.373 - 0.586 | 0.333 - 0.676 | 0.053 |  |
| *period* | 5 d reactivation | vs. | revived 2 | 2.086 | 1.512 - 2.749 | 1.327 - 3.593 | 0.033 | UP |
| *period* | 5 d reactivation | vs. | revived 3 | 1.226 | 0.978 - 1.603 | 0.808 - 1.861 | 0.419 |  |
| *period* | 12 d reactivation | vs. | 19 d reactivation | 0.939 | 0.740 - 1.122 | 0.705 - 1.410 | 0.796 |  |
| *period* | 12 d reactivation | vs. | revived 1 | 0.431 | 0.366 - 0.503 | 0.340 - 0.581 | 0 | DOWN |
| *period* | 12 d reactivation | vs. | revived 2 | 1.917 | 1.465 - 2.467 | 1.347 - 3.100 | 0 | UP |
| *period* | 12 d reactivation | vs. | revived 3 | 1.127 | 0.902 - 1.387 | 0.798 - 1.599 | 0.33 |  |
| *period* | 19 d reactivation | vs. | revived 1 | 0.459 | 0.364 - 0.564 | 0.325 - 0.600 | 0.029 | DOWN |
| *period* | 19 d reactivation | vs. | revived 2 | 2.041 | 1.534 - 2.893 | 1.296 - 3.258 | 0 | UP |
| *period* | 19 d reactivation | vs. | revived 3 | 1.2 | 0.996 - 1.555 | 0.793 - 1.649 | 0.232 |  |
| *period* | revived 1 | vs. | revived 2 | 4.447 | 3.556 - 6.061 | 3.204 - 6.765 | 0 | UP |
| *period* | revived 1 | vs. | revived 3 | 2.614 | 2.199 - 3.143 | 1.909 - 3.423 | 0.049 | UP |
| *period* | revived 2 | vs. | revived 3 | 0.588 | 0.458 - 0.775 | 0.361 - 0.860 | 0.028 | DOWN |
| *cry2* | 24 h | vs. | 48 h | 1.958 | 1.790 - 2.204 | 1.647 - 2.288 | 0 | UP |
| *cry2* | 24 h | vs. | 74 h | 1.539 | 1.250 - 1.834 | 1.119 - 1.848 | 0.046 | UP |
| *cry2* | 24 h | vs. | 1 m dormant | 1.913 | 1.745 - 2.172 | 1.562 - 2.226 | 0.017 | UP |
| *cry2* | 24 h | vs. | 11 m dormant | 0.633 | 0.495 - 0.862 | 0.443 - 0.904 | 0 | DOWN |
| *cry2* | 24 h | vs. | 1 d reactivation | 1.72 | 1.267 - 2.926 | 1.178 - 3.067 | 0 | UP |
| *cry2* | 24 h | vs. | 5 d reactivation | 1.074 | 0.869 - 1.321 | 0.778 - 1.382 | 0.522 |  |
| *cry2* | 24 h | vs. | 12 d reactivation | 0.607 | 0.480 - 0.750 | 0.430 - 0.780 | 0 | DOWN |
| *cry2* | 24 h | vs. | 19 d reactivation | 0.64 | 0.548 - 0.815 | 0.491 - 0.855 | 0 | DOWN |
| *cry2* | 24 h | vs. | revived 1 | 1.838 | 1.684 - 2.059 | 1.507 - 2.084 | 0.03 | UP |
| *cry2* | 24 h | vs. | revived 2 | 7.73 | 6.007 - 9.764 | 5.377 - 10.234 | 0 | UP |
| *cry2* | 24 h | vs. | revived 3 | 9.504 | 6.740 - 13.536 | 6.033 - 14.188 | 0 | UP |
| *cry2* | 48 h | vs. | 74 h | 0.786 | 0.631 - 0.957 | 0.564 - 0.983 | 0 | DOWN |
| *cry2* | 48 h | vs. | 1 m dormant | 0.977 | 0.874 - 1.109 | 0.787 - 1.174 | 0.772 |  |
| *cry2* | 48 h | vs. | 11 m dormant | 0.323 | 0.250 - 0.429 | 0.223 - 0.474 | 0 | DOWN |
| *cry2* | 48 h | vs. | 1 d reactivation | 0.878 | 0.637 - 1.455 | 0.597 - 1.609 | 0.604 |  |
| *cry2* | 48 h | vs. | 5 d reactivation | 0.549 | 0.439 - 0.665 | 0.392 - 0.725 | 0 | DOWN |
| *cry2* | 48 h | vs. | 12 d reactivation | 0.31 | 0.242 - 0.378 | 0.216 - 0.409 | 0 | DOWN |
| *cry2* | 48 h | vs. | 19 d reactivation | 0.327 | 0.274 - 0.406 | 0.247 - 0.448 | 0 | DOWN |
| *cry2* | 48 h | vs. | revived 1 | 0.939 | 0.847 - 1.074 | 0.759 - 1.109 | 0.492 |  |
| *cry2* | 48 h | vs. | revived 2 | 3.948 | 3.032 - 4.905 | 2.709 - 5.370 | 0.062 |  |
| *cry2* | 48 h | vs. | revived 3 | 4.854 | 3.402 - 6.733 | 3.039 - 7.444 | 0.085 |  |
| *cry2* | 74 h | vs. | 1 m dormant | 1.243 | 1.007 - 1.589 | 0.955 - 1.746 | 0.193 |  |
| *cry2* | 74 h | vs. | 11 m dormant | 0.411 | 0.295 - 0.514 | 0.271 - 0.678 | 0.051 |  |
| *cry2* | 74 h | vs. | 1 d reactivation | 1.118 | 0.747 - 1.670 | 0.731 - 2.282 | 0.686 |  |
| *cry2* | 74 h | vs. | 5 d reactivation | 0.698 | 0.524 - 0.897 | 0.475 - 1.068 | 0.106 |  |
| *cry2* | 74 h | vs. | 12 d reactivation | 0.394 | 0.294 - 0.516 | 0.263 - 0.605 | 0 | DOWN |
| *cry2* | 74 h | vs. | 19 d reactivation | 0.416 | 0.315 - 0.502 | 0.300 - 0.646 | 0 | DOWN |
| *cry2* | 74 h | vs. | revived 1 | 1.194 | 0.976 - 1.552 | 0.921 - 1.649 | 0.225 |  |
| *cry2* | 74 h | vs. | revived 2 | 5.024 | 3.690 - 6.555 | 3.287 - 7.883 | 0 | UP |
| *cry2* | 74 h | vs. | revived 3 | 6.177 | 4.165 - 8.123 | 3.688 - 10.663 | 0 | UP |
| *cry2* | 1 m dormant | vs. | 11 m dormant | 0.331 | 0.251 - 0.425 | 0.227 - 0.498 | 0.016 | DOWN |
| *cry2* | 1 m dormant | vs. | 1 d reactivation | 0.899 | 0.645 - 1.442 | 0.613 - 1.688 | 0.707 |  |
| *cry2* | 1 m dormant | vs. | 5 d reactivation | 0.561 | 0.440 - 0.675 | 0.398 - 0.765 | 0 | DOWN |
| *cry2* | 1 m dormant | vs. | 12 d reactivation | 0.317 | 0.243 - 0.388 | 0.220 - 0.434 | 0 | DOWN |
| *cry2* | 1 m dormant | vs. | 19 d reactivation | 0.334 | 0.272 - 0.402 | 0.251 - 0.471 | 0 | DOWN |
| *cry2* | 1 m dormant | vs. | revived 1 | 0.96 | 0.841 - 1.120 | 0.772 - 1.181 | 0.416 |  |
| *cry2* | 1 m dormant | vs. | revived 2 | 4.04 | 3.043 - 4.938 | 2.755 - 5.647 | 0.075 |  |
| *cry2* | 1 m dormant | vs. | revived 3 | 4.967 | 3.415 - 6.671 | 3.091 - 7.811 | 0.064 |  |
| *cry2* | 11 m dormant | vs. | 1 d reactivation | 2.717 | 1.717 - 4.319 | 1.510 - 5.848 | 0 | UP |
| *cry2* | 11 m dormant | vs. | 5 d reactivation | 1.697 | 1.326 - 2.318 | 1.040 - 2.695 | 0.166 |  |
| *cry2* | 11 m dormant | vs. | 12 d reactivation | 0.959 | 0.744 - 1.333 | 0.575 - 1.528 | 0.8 |  |
| *cry2* | 11 m dormant | vs. | 19 d reactivation | 1.011 | 0.751 - 1.300 | 0.632 - 1.630 | 0.898 |  |
| *cry2* | 11 m dormant | vs. | revived 1 | 2.903 | 2.279 - 3.916 | 1.956 - 4.163 | 0.041 | UP |
| *cry2* | 11 m dormant | vs. | revived 2 | 12.211 | 9.328 - 16.946 | 7.201 - 19.896 | 0 | UP |
| *cry2* | 11 m dormant | vs. | revived 3 | 15.014 | 10.658 - 21.389 | 8.079 - 27.058 | 0.046 | UP |
| *cry2* | 1 d reactivation | vs. | 5 d reactivation | 0.625 | 0.407 - 0.940 | 0.307 - 1.013 | 0.111 |  |
| *cry2* | 1 d reactivation | vs. | 12 d reactivation | 0.353 | 0.234 - 0.534 | 0.171 - 0.572 | 0 | DOWN |
| *cry2* | 1 d reactivation | vs. | 19 d reactivation | 0.372 | 0.230 - 0.563 | 0.186 - 0.626 | 0 | DOWN |
| *cry2* | 1 d reactivation | vs. | revived 1 | 1.068 | 0.672 - 1.502 | 0.576 - 1.543 | 0.832 |  |
| *cry2* | 1 d reactivation | vs. | revived 2 | 4.495 | 2.977 - 6.934 | 2.148 - 7.501 | 0 | UP |
| *cry2* | 1 d reactivation | vs. | revived 3 | 5.526 | 3.731 - 9.275 | 2.451 - 10.399 | 0 | UP |
| *cry2* | 5 d reactivation | vs. | 12 d reactivation | 0.565 | 0.431 - 0.753 | 0.361 - 0.871 | 0.082 |  |
| *cry2* | 5 d reactivation | vs. | 19 d reactivation | 0.596 | 0.464 - 0.734 | 0.412 - 0.929 | 0.07 |  |
| *cry2* | 5 d reactivation | vs. | revived 1 | 1.711 | 1.436 - 2.232 | 1.266 - 2.373 | 0.023 | UP |
| *cry2* | 5 d reactivation | vs. | revived 2 | 7.197 | 5.397 - 9.570 | 4.516 - 11.340 | 0 | UP |
| *cry2* | 5 d reactivation | vs. | revived 3 | 8.848 | 6.181 - 11.880 | 5.067 - 15.340 | 0 | UP |
| *cry2* | 12 d reactivation | vs. | 19 d reactivation | 1.054 | 0.807 - 1.312 | 0.726 - 1.682 | 0.696 |  |
| *cry2* | 12 d reactivation | vs. | revived 1 | 3.028 | 2.498 - 4.041 | 2.231 - 4.295 | 0.039 | UP |
| *cry2* | 12 d reactivation | vs. | revived 2 | 12.736 | 9.401 - 17.133 | 7.958 - 20.529 | 0 | UP |
| *cry2* | 12 d reactivation | vs. | revived 3 | 15.659 | 10.770 - 21.242 | 8.929 - 27.770 | 0 | UP |
| *cry2* | 19 d reactivation | vs. | revived 1 | 2.872 | 2.410 - 3.576 | 2.068 - 3.761 | 0.037 | UP |
| *cry2* | 19 d reactivation | vs. | revived 2 | 12.079 | 9.566 - 15.773 | 7.534 - 17.974 | 0 | UP |
| *cry2* | 19 d reactivation | vs. | revived 3 | 14.851 | 10.936 - 21.045 | 8.453 - 24.918 | 0 | UP |
| *cry2* | revived 1 | vs. | revived 2 | 4.207 | 3.120 - 5.096 | 2.914 - 5.852 | 0 | UP |
| *cry2* | revived 1 | vs. | revived 3 | 5.172 | 3.500 - 6.887 | 3.270 - 8.072 | 0 | UP |
| *cry2* | revived 2 | vs. | revived 3 | 1.229 | 0.844 - 1.695 | 0.687 - 2.219 | 0.308 |  |
| *brp* | 24 h | vs. | 48 h | 1.719 | 1.474 - 2.070 | 1.339 - 2.221 | 0.06 |  |
| *brp* | 24 h | vs. | 74 h | 2.201 | 1.705 - 2.909 | 1.460 - 3.121 | 0.062 |  |
| *brp* | 24 h | vs. | 1 m dormant | 3.368 | 3.040 - 3.744 | 2.810 - 3.783 | 0.037 | UP |
| *brp* | 24 h | vs. | 11 m dormant | 0.591 | 0.458 - 0.852 | 0.429 - 0.914 | 0.01 | DOWN |
| *brp* | 24 h | vs. | 1 d reactivation | 1.991 | 1.726 - 2.356 | 1.569 - 2.516 | 0.079 |  |
| *brp* | 24 h | vs. | 5 d reactivation | 1.235 | 0.893 - 2.054 | 0.831 - 2.203 | 0.574 |  |
| *brp* | 24 h | vs. | 12 d reactivation | 0.567 | 0.417 - 0.930 | 0.379 - 0.997 | 0.107 |  |
| *brp* | 24 h | vs. | 19 d reactivation | 0.54 | 0.388 - 0.832 | 0.332 - 0.892 | 0 | DOWN |
| *brp* | 24 h | vs. | revived 1 | 2.436 | 2.091 - 2.997 | 1.849 - 3.215 | 0.072 |  |
| *brp* | 24 h | vs. | revived 2 | 10.635 | 8.968 - 13.005 | 8.337 - 13.950 | 0.07 |  |
| *brp* | 24 h | vs. | revived 3 | 16.463 | 14.473 - 18.499 | 13.652 - 19.338 | 0.074 |  |
| *brp* | 48 h | vs. | 74 h | 1.281 | 1.003 - 1.701 | 0.819 - 1.892 | 0.263 |  |
| *brp* | 48 h | vs. | 1 m dormant | 1.96 | 1.690 - 2.260 | 1.561 - 2.318 | 0.012 | UP |
| *brp* | 48 h | vs. | 11 m dormant | 0.344 | 0.258 - 0.488 | 0.239 - 0.554 | 0.036 | DOWN |
| *brp* | 48 h | vs. | 1 d reactivation | 1.159 | 0.971 - 1.383 | 0.872 - 1.525 | 0.223 |  |
| *brp* | 48 h | vs. | 5 d reactivation | 0.719 | 0.502 - 1.177 | 0.462 - 1.335 | 0.197 |  |
| *brp* | 48 h | vs. | 12 d reactivation | 0.33 | 0.235 - 0.533 | 0.211 - 0.604 | 0 | DOWN |
| *brp* | 48 h | vs. | 19 d reactivation | 0.314 | 0.228 - 0.477 | 0.186 - 0.541 | 0 | DOWN |
| *brp* | 48 h | vs. | revived 1 | 1.417 | 1.176 - 1.751 | 1.027 - 1.949 | 0.138 |  |
| *brp* | 48 h | vs. | revived 2 | 6.188 | 5.046 - 7.522 | 4.633 - 8.455 | 0 | UP |
| *brp* | 48 h | vs. | revived 3 | 9.579 | 8.042 - 10.866 | 7.586 - 11.721 | 0 | UP |
| *brp* | 74 h | vs. | 1 m dormant | 1.53 | 1.202 - 2.110 | 1.111 - 2.174 | 0.016 | UP |
| *brp* | 74 h | vs. | 11 m dormant | 0.269 | 0.183 - 0.353 | 0.170 - 0.496 | 0.071 |  |
| *brp* | 74 h | vs. | 1 d reactivation | 0.905 | 0.691 - 1.169 | 0.621 - 1.402 | 0.647 |  |
| *brp* | 74 h | vs. | 5 d reactivation | 0.561 | 0.358 - 0.839 | 0.328 - 1.196 | 0.106 |  |
| *brp* | 74 h | vs. | 12 d reactivation | 0.258 | 0.167 - 0.380 | 0.150 - 0.541 | 0.043 | DOWN |
| *brp* | 74 h | vs. | 19 d reactivation | 0.245 | 0.165 - 0.351 | 0.133 - 0.484 | 0 | DOWN |
| *brp* | 74 h | vs. | revived 1 | 1.107 | 0.837 - 1.424 | 0.731 - 1.780 | 0.529 |  |
| *brp* | 74 h | vs. | revived 2 | 4.832 | 3.593 - 6.063 | 3.296 - 7.674 | 0 | UP |
| *brp* | 74 h | vs. | revived 3 | 7.479 | 5.722 - 9.910 | 5.398 - 10.939 | 0 | UP |
| *brp* | 1 m dormant | vs. | 11 m dormant | 0.176 | 0.141 - 0.244 | 0.140 - 0.264 | 0.011 | DOWN |
| *brp* | 1 m dormant | vs. | 1 d reactivation | 0.591 | 0.519 - 0.671 | 0.506 - 0.727 | 0 | DOWN |
| *brp* | 1 m dormant | vs. | 5 d reactivation | 0.367 | 0.274 - 0.587 | 0.271 - 0.636 | 0 | DOWN |
| *brp* | 1 m dormant | vs. | 12 d reactivation | 0.168 | 0.125 - 0.266 | 0.122 - 0.288 | 0 | DOWN |
| *brp* | 1 m dormant | vs. | 19 d reactivation | 0.16 | 0.108 - 0.238 | 0.105 - 0.258 | 0 | DOWN |
| *brp* | 1 m dormant | vs. | revived 1 | 0.723 | 0.608 - 0.857 | 0.589 - 0.929 | 0 | DOWN |
| *brp* | 1 m dormant | vs. | revived 2 | 3.158 | 2.749 - 3.718 | 2.714 - 4.030 | 0.075 |  |
| *brp* | 1 m dormant | vs. | revived 3 | 4.888 | 4.501 - 5.155 | 4.448 - 5.586 | 0.064 |  |
| *brp* | 11 m dormant | vs. | 1 d reactivation | 3.367 | 2.396 - 4.470 | 2.119 - 4.749 | 0.061 |  |
| *brp* | 11 m dormant | vs. | 5 d reactivation | 2.088 | 1.354 - 3.636 | 1.122 - 4.158 | 0.07 |  |
| *brp* | 11 m dormant | vs. | 12 d reactivation | 0.959 | 0.631 - 1.646 | 0.512 - 1.882 | 0.695 |  |
| *brp* | 11 m dormant | vs. | 19 d reactivation | 0.913 | 0.647 - 1.497 | 0.468 - 1.684 | 0.842 |  |
| *brp* | 11 m dormant | vs. | revived 1 | 4.119 | 2.985 - 5.663 | 2.496 - 6.069 | 0.056 |  |
| *brp* | 11 m dormant | vs. | revived 2 | 17.984 | 12.592 - 24.341 | 11.254 - 26.333 | 0.032 | UP |
| *brp* | 11 m dormant | vs. | revived 3 | 27.839 | 19.537 - 35.089 | 18.429 - 36.502 | 0.031 | UP |
| *brp* | 1 d reactivation | vs. | 5 d reactivation | 0.62 | 0.439 - 1.014 | 0.408 - 1.139 | 0.203 |  |
| *brp* | 1 d reactivation | vs. | 12 d reactivation | 0.285 | 0.205 - 0.459 | 0.186 - 0.515 | 0.041 | DOWN |
| *brp* | 1 d reactivation | vs. | 19 d reactivation | 0.271 | 0.195 - 0.410 | 0.163 - 0.461 | 0.033 | DOWN |
| *brp* | 1 d reactivation | vs. | revived 1 | 1.223 | 1.028 - 1.496 | 0.907 - 1.662 | 0.131 |  |
| *brp* | 1 d reactivation | vs. | revived 2 | 5.342 | 4.410 - 6.425 | 4.089 - 7.212 | 0 | UP |
| *brp* | 1 d reactivation | vs. | revived 3 | 8.269 | 7.099 - 9.280 | 6.696 - 9.997 | 0 | UP |
| *brp* | 5 d reactivation | vs. | 12 d reactivation | 0.459 | 0.284 - 0.822 | 0.212 - 0.973 | 0.082 |  |
| *brp* | 5 d reactivation | vs. | 19 d reactivation | 0.437 | 0.288 - 0.766 | 0.194 - 0.870 | 0.033 | DOWN |
| *brp* | 5 d reactivation | vs. | revived 1 | 1.972 | 1.239 - 2.901 | 1.036 - 3.137 | 0.023 | UP |
| *brp* | 5 d reactivation | vs. | revived 2 | 8.612 | 5.225 - 12.466 | 4.670 - 13.610 | 0 | UP |
| *brp* | 5 d reactivation | vs. | revived 3 | 13.331 | 8.108 - 17.979 | 7.648 - 18.866 | 0 | UP |
| *brp* | 12 d reactivation | vs. | 19 d reactivation | 0.952 | 0.625 - 1.632 | 0.429 - 1.909 | 0.799 |  |
| *brp* | 12 d reactivation | vs. | revived 1 | 4.293 | 2.736 - 6.188 | 2.288 - 6.882 | 0.039 | UP |
| *brp* | 12 d reactivation | vs. | revived 2 | 18.745 | 11.543 - 26.582 | 10.317 - 29.861 | 0 | UP |
| *brp* | 12 d reactivation | vs. | revived 3 | 29.017 | 17.910 - 38.395 | 16.894 - 41.393 | 0 | UP |
| *brp* | 19 d reactivation | vs. | revived 1 | 4.51 | 3.058 - 6.260 | 2.557 - 7.823 | 0.037 | UP |
| *brp* | 19 d reactivation | vs. | revived 2 | 19.691 | 12.902 - 26.640 | 11.532 - 33.717 | 0 | UP |
| *brp* | 19 d reactivation | vs. | revived 3 | 30.482 | 20.019 - 43.542 | 18.884 - 48.066 | 0 | UP |
| *brp* | revived 1 | vs. | revived 2 | 4.366 | 3.490 - 5.290 | 3.200 - 6.134 | 0 | UP |
| *brp* | revived 1 | vs. | revived 3 | 6.759 | 5.555 - 7.754 | 5.240 - 8.547 | 0.033 | UP |
| *brp* | revived 2 | vs. | revived 3 | 1.548 | 1.280 - 1.789 | 1.208 - 1.880 | 0 | UP |

**Table S2:** Fold gene expression change and statistical results of sexually produced *Daphnia* embryos.

| **gene** | **Control stage** | **vs.** | **Treatment stage** | **fold expression** | **standard error** | **95% confidence interval** | **pValue** | **results** |
| --- | --- | --- | --- | --- | --- | --- | --- | --- |
| *clock* | 10 h | vs. | 15 h | 0.873 | 0.662 - 1.108 | 0.593 - 1.431 | 0.67 |  |
| *clock* | 10 h | vs. | 24 h | 0.962 | 0.824 - 1.209 | 0.778 - 1.238 | 0.684 |  |
| *clock* | 10 h | vs. | red-eye stage | 1.585 | 1.377 - 1.997 | 1.240 - 2.090 | 0 | UP |
| *clock* | 10 h | vs. | black-eye stage | 2.215 | 1.844 - 2.669 | 1.687 - 3.070 | 0 | UP |
| *clock* | 15 h | vs. | 24 h | 1.102 | 0.821 - 1.400 | 0.776 - 1.432 | 0.603 |  |
| *clock* | 15 h | vs. | red-eye stage | 1.814 | 1.381 - 2.312 | 1.237 - 2.419 | 0 | UP |
| *clock* | 15 h | vs. | black-eye stage | 2.535 | 1.898 - 3.159 | 1.681 - 3.553 | 0 | UP |
| *clock* | 24 h | vs. | red-eye stage | 1.647 | 1.496 - 1.773 | 1.460 - 1.818 | 0 | UP |
| *clock* | 24 h | vs. | black-eye stage | 2.301 | 2.047 - 2.555 | 1.998 - 2.706 | 0 | UP |
| *clock* | red-eye stage | vs. | black-eye stage | 1.397 | 1.226 - 1.522 | 1.183 - 1.700 | 0.052 |  |
| *cycle* | 10 h | vs. | 15 h | 1.001 | 0.815 - 1.200 | 0.742 - 1.436 | 0.905 |  |
| *cycle* | 10 h | vs. | 24 h | 1.328 | 1.135 - 1.554 | 1.112 - 1.626 | 0.103 |  |
| *cycle* | 10 h | vs. | red-eye stage | 4.817 | 4.265 - 5.730 | 3.855 - 6.067 | 0 | UP |
| *cycle* | 10 h | vs. | black-eye stage | 9.474 | 8.407 - 11.342 | 7.671 - 11.771 | 0 | UP |
| *cycle* | 15 h | vs. | 24 h | 1.327 | 1.062 - 1.539 | 1.040 - 1.610 | 0.048 | UP |
| *cycle* | 15 h | vs. | red-eye stage | 4.813 | 4.008 - 5.738 | 3.607 - 6.007 | 0 | UP |
| *cycle* | 15 h | vs. | black-eye stage | 9.466 | 7.866 - 11.266 | 7.177 - 11.655 | 0 | UP |
| *cycle* | 24 h | vs. | red-eye stage | 3.628 | 3.367 - 3.894 | 3.221 - 3.942 | 0 | UP |
| *cycle* | 24 h | vs. | black-eye stage | 7.136 | 6.715 - 7.567 | 6.423 - 7.615 | 0.046 | UP |
| *cycle* | red-eye stage | vs. | black-eye stage | 1.967 | 1.805 - 2.167 | 1.721 - 2.249 | 0.068 |  |
| *tim* | 10 h | vs. | 15 h | 0.993 | 0.779 - 1.355 | 0.718 - 1.504 | 0.949 |  |
| *tim* | 10 h | vs. | 24 h | 0.452 | 0.425 - 0.497 | 0.406 - 0.506 | 0 | DOWN |
| *tim* | 10 h | vs. | red-eye stage | 0.835 | 0.695 - 0.969 | 0.640 - 1.069 | 0.152 |  |
| *tim* | 10 h | vs. | black-eye stage | 0.998 | 0.924 - 1.079 | 0.893 - 1.134 | 1 |  |
| *tim* | 15 h | vs. | 24 h | 0.455 | 0.329 - 0.591 | 0.314 - 0.601 | 0.093 |  |
| *tim* | 15 h | vs. | red-eye stage | 0.841 | 0.640 - 1.110 | 0.514 - 1.261 | 0.399 |  |
| *tim* | 15 h | vs. | black-eye stage | 1.005 | 0.719 - 1.282 | 0.692 - 1.347 | 0.955 |  |
| *tim* | 24 h | vs. | red-eye stage | 1.849 | 1.510 - 2.160 | 1.483 - 2.263 | 0 | UP |
| *tim* | 24 h | vs. | black-eye stage | 2.21 | 2.106 - 2.278 | 2.086 - 2.378 | 0.046 | UP |
| *tim* | red-eye stage | vs. | black-eye stage | 1.195 | 1.011 - 1.438 | 0.973 - 1.511 | 0.248 |  |
| *period* | 10 h | vs. | 15 h | 0.495 | 0.364 - 0.613 | 0.332 - 0.704 | 0.096 |  |
| *period* | 10 h | vs. | 24 h | 0.527 | 0.397 - 0.659 | 0.347 - 0.749 | 0 | DOWN |
| *period* | 10 h | vs. | red-eye stage | 4.393 | 3.278 - 5.435 | 3.052 - 5.847 | 0 | UP |
| *period* | 10 h | vs. | black-eye stage | 4.152 | 3.162 - 5.244 | 2.863 - 5.495 | 0 | UP |
| *period* | 15 h | vs. | 24 h | 1.064 | 0.922 - 1.262 | 0.824 - 1.348 | 0.495 |  |
| *period* | 15 h | vs. | red-eye stage | 8.879 | 7.783 - 9.963 | 7.247 - 10.366 | 0 | UP |
| *period* | 15 h | vs. | black-eye stage | 8.392 | 7.508 - 9.484 | 6.798 - 9.687 | 0 | UP |
| *period* | 24 h | vs. | red-eye stage | 8.342 | 7.316 - 9.379 | 6.812 - 10.019 | 0 | UP |
| *period* | 24 h | vs. | black-eye stage | 7.885 | 7.057 - 9.056 | 6.389 - 9.417 | 0.095 |  |
| *period* | red-eye stage | vs. | black-eye stage | 0.945 | 0.880 - 1.032 | 0.822 - 1.061 | 0.298 |  |
| *cry2* | 10 h | vs. | 15 h | 1.202 | 1.027 - 1.473 | 0.876 - 1.615 | 0.112 |  |
| *cry2* | 10 h | vs. | 24 h | 1.093 | 0.921 - 1.283 | 0.834 - 1.430 | 0.562 |  |
| *cry2* | 10 h | vs. | red-eye stage | 3.271 | 2.560 - 4.165 | 2.252 - 5.032 | 0 | UP |
| *cry2* | 10 h | vs. | black-eye stage | 9.163 | 7.550 - 10.626 | 7.177 - 11.792 | 0 | UP |
| *cry2* | 15 h | vs. | 24 h | 0.909 | 0.794 - 1.041 | 0.744 - 1.164 | 0.357 |  |
| *cry2* | 15 h | vs. | red-eye stage | 2.722 | 2.153 - 3.368 | 1.984 - 4.078 | 0 | UP |
| *cry2* | 15 h | vs. | black-eye stage | 7.623 | 6.623 - 8.651 | 6.407 - 9.601 | 0 | UP |
| *cry2* | 24 h | vs. | red-eye stage | 2.993 | 2.470 - 3.867 | 2.243 - 4.266 | 0 | UP |
| *cry2* | 24 h | vs. | black-eye stage | 8.384 | 7.503 - 9.143 | 7.199 - 9.894 | 0.064 |  |
| *cry2* | red-eye stage | vs. | black-eye stage | 2.801 | 2.125 - 3.319 | 2.020 - 3.683 | 0 | UP |
| *brp* | 10 h | vs. | 15 h | 0.89 | 0.683 - 1.134 | 0.588 - 1.409 | 0.594 |  |
| *brp* | 10 h | vs. | 24 h | 0.989 | 0.793 - 1.285 | 0.680 - 1.474 | 0.806 |  |
| *brp* | 10 h | vs. | red-eye stage | 3.901 | 2.883 - 4.967 | 2.519 - 6.494 | 0 | UP |
| *brp* | 10 h | vs. | black-eye stage | 6.468 | 5.069 - 8.457 | 4.784 - 9.012 | 0 | UP |
| *brp* | 15 h | vs. | 24 h | 1.112 | 0.930 - 1.358 | 0.794 - 1.512 | 0.495 |  |
| *brp* | 15 h | vs. | red-eye stage | 4.383 | 3.381 - 5.672 | 2.943 - 6.820 | 0 | UP |
| *brp* | 15 h | vs. | black-eye stage | 7.268 | 5.922 - 8.675 | 5.589 - 9.244 | 0.048 | UP |
| *brp* | 24 h | vs. | red-eye stage | 3.943 | 3.153 - 4.956 | 2.863 - 5.898 | 0 | UP |
| *brp* | 24 h | vs. | black-eye stage | 6.538 | 5.792 - 7.512 | 5.466 - 8.005 | 0.046 | UP |
| *brp* | red-eye stage | vs. | black-eye stage | 1.658 | 1.271 - 2.009 | 1.199 - 2.141 | 0 | UP |

**Table S3:** List of mean Cqs per measured gene and treatment

| embryo type | stage | *brp* | *clk* | *cry2* | *cyc* | *per* | *tbp* | *tim* |
| --- | --- | --- | --- | --- | --- | --- | --- | --- |
| sexually produced embryo | 24h | 27.85 | 22.995 | 25.45 | 25.335 | 21.335 | 20.36 | 21.38 |
|  | 24h | 27.885 | 23.035 | 25.85 | 25.325 | 22.025 | 20.74 | 21.87 |
|  | 24h | 28.41 | 22.98 | 25.84 | 25.42 | 21.595 | 20.97 | 21.475 |
|  |  |  |  |  |  |  |  |  |
|  | 48h | 28.56 | 23.86 | 26.14 | 24.755 | 22.355 | 22.36 | 22.46 |
|  | 48h | 28.515 | 23.475 | 26.05 | 24.715 | 22.575 | 21.95 | 22.225 |
|  | 48h | 28.655 | 23.805 | 25.915 | 24.75 | 22.325 | 21.955 | 22.41 |
|  |  |  |  |  |  |  |  |  |
|  | 74h | 27.09 | 23.74 | 25.56 | 23.985 | 22.79 | 21.42 | 22.245 |
|  | 74h | 27.84 | 23.89 | 25.97 | 24.275 | 22.735 | 21.235 | 22.54 |
|  | 74h | 27.475 | 24.3 | 25.57 | 24.075 | 22.71 | 21.44 | 22.72 |
|  |  |  |  |  |  |  |  |  |
|  | 1 m dormant | 26.895 | 28.31 | 25.445 | 24.36 | 22.455 | 21.515 | 22.31 |
|  | 1 m dormant | 27.015 | 23.635 | 25.685 | 24.84 | 22.665 | 21.48 | 22.495 |
|  | 1 m dormant | 27.08 | 23.645 | 25.54 | 25.01 | 22.355 | 21.71 | 22.45 |
|  |  |  |  |  |  |  |  |  |
|  | 11 m dormant | 29.15 | 24.49 | 26.865 | 26.165 | 24.045 | 20.66 | 23.43 |
|  | 11 m dormant | 29.09 | 24.16 | 26.4 | 25.785 | 23.84 | 20.63 | 23.23 |
|  | 11 m dormant | 28.345 | 24.38 | 25.99 | 25.585 | 23.725 | 20.725 | 23.06 |
|  |  |  |  |  |  |  |  |  |
|  | 1 d reactivation | 27.555 | 23.585 | 24.87 | 24.225 | 22.03 | 21.545 | 22.42 |
|  | 1 d reactivation | 28.13 | 23.66 | 26.305 | 24.39 | 21.605 | 21.68 | 22.585 |
|  | 1 d reactivation | 27.89 | 23.535 | 26.09 | 24.245 | 21.59 | 21.57 | 22.08 |
|  |  |  |  |  |  |  |  |  |
|  | 5 d reactivation | 29.735 | 24.465 | 27.105 | 24.75 | 22.075 | 22.3 | 22.955 |
|  | 5 d reactivation | 27.8 | 24.06 | 26.165 | 24.48 | 21.74 | 21.58 | 22.605 |
|  | 5 d reactivation | 29.125 | 24.485 | 27.045 | 24.635 | 22.34 | 21.74 | 22.89 |
|  |  |  |  |  |  |  |  |  |
|  | 12 d reactivation | 30.345 | 24.66 | 27.455 | 24.81 | 21.745 | 21.78 | 23.06 |
|  | 12 d reactivation | 30.305 | 24.55 | 27.85 | 24.93 | 21.965 | 21.605 | 23.09 |
|  | 12 d reactivation | 29.525 | 24.92 | 27.54 | 24.96 | 21.865 | 22.055 | 23.08 |
|  |  |  |  |  |  |  |  |  |
|  | 19 d reactivation | 29.965 | 24.02 | 27.62 | 24.9 | 21.965 | 21.585 | 22.58 |
|  | 19 d reactivation | 29.3 | 24.315 | 27.43 | 25.15 | 21.965 | 21.65 | 22.8 |
|  | 19 d reactivation | 31.02 | 24.35 | 27.42 | 25.195 | 21.82 | 22.08 | 22.845 |
|  |  |  |  |  |  |  |  |  |
|  | revived 1 | 26.29 | 22.195 | 24.63 | 23.925 | 21.845 | 20.66 | 22.1 |
|  | revived 1 | 27.105 | 22.925 | 25.05 | 24.525 | 22.525 | 21.11 | 22.67 |
|  | revived 1 | 26.85 | 22.575 | 24.9 | 24.19 | 22.19 | 20.63 | 22.08 |
|  |  |  |  |  |  |  |  |  |
|  | revived 2 | 26.715 | 22.8 | 24.34 | 23.43 | 21.42 | 22.935 | 22.25 |
|  | revived 2 | 24.185 | 21.49 | 22.56 | 22.19 | 20.26 | 20.88 | 21.05 |
|  | revived 2 | 25.685 | 22.58 | 24.2 | 23.485 | 21.13 | 21.955 | 22.335 |
|  |  |  |  |  |  |  |  |  |
|  | revived 3 | 25.21 | 24.11 | 23.59 | 23.575 | 21.775 | 22.205 | 22.9 |
|  | revived 3 | 24.535 | 22.775 | 23.82 | 22.585 | 21.55 | 21.755 | 22.695 |
|  | revived 3 | 24.255 | 22.13 | 22.2 | 22.04 | 21.5 | 21.295 | 21.775 |
|  |  |  |  |  |  |  |  |  |
| asexually produced embryo | 10h | 27.4 | 23.235 | 25.8 | 25.61 | 23.815 | 21.37 | 21.805 |
|  | 10h | 28 | 23.77 | 26.19 | 26.02 | 24.16 | 21.475 | 22.08 |
|  | 10h | 27.255 | 23.245 | 25.7 | 25.61 | 23.415 | 21.59 | 21.92 |
|  |  |  |  |  |  |  |  |  |
|  | 15h | 27.865 | 23.22 | 25.63 | 25.485 | 24.765 | 21.57 | 21.495 |
|  | 15h | 27.535 | 23.25 | 25.36 | 25.5 | 24.49 | 20.965 | 21.57 |
|  | 15h | 26.56 | 23.175 | 24.58 | 25.005 | 24.23 | 20.64 | 21.525 |
|  |  |  |  |  |  |  |  |  |
|  | 24h | 26.63 | 22.65 | 24.69 | 24.42 | 23.94 | 20.565 | 22.26 |
|  | 4h | 27.01 | 22.63 | 25.1 | 24.53 | 24.21 | 20.665 | 22.355 |
|  | 4h | 26.5 | 22.59 | 24.915 | 24.385 | 23.72 | 20.615 | 22.4 |
|  |  |  |  |  |  |  |  |  |
|  | red-eye stage | 25.095 | 22.765 | 23.725 | 23.305 | 21.485 | 21.62 | 22.085 |
|  | red-eye stage | 25.605 | 22.7 | 24.225 | 23.31 | 21.55 | 21.575 | 22.275 |
|  | red-eye stage | 25.7 | 22.81 | 24.325 | 23.38 | 21.53 | 21.45 | 22.51 |
|  |  |  |  |  |  |  |  |  |
|  | black-eye stage | 25.225 | 22.5 | 23.02 | 22.665 | 22 | 22.025 | 22.41 |
|  | black-eye stage | 24.61 | 22.285 | 22.36 | 22.245 | 21.595 | 21.585 | 22.055 |
|  | black-eye stage | 24.515 | 22.255 | 22.36 | 22.23 | 21.59 | 21.39 | 21.91 |
